# Anti-inflammatory sesquiterpene and triterpene acids from *Mesona procumbens* Hemsley

**DOI:** 10.3389/fchem.2022.1003356

**Published:** 2022-09-15

**Authors:** Hung-Tse Huang, I-Wen Lo, Geng-You Liao, Yu-Chi Lin, Yuh-Chiang Shen, Hui-Chi Huang, Tsung-Lin Li, Kung-Ta Lee, Yao-Haur Kuo, Chia-Ching Liaw

**Affiliations:** ^1^ National Research Institute of Chinese Medicine, Taipei, Taiwan; ^2^ Department of Biochemical Science and Technology, National Taiwan University, Taipei, Taiwan; ^3^ Genomics Research Center, Academia Sinica, Taipei, Taiwan; ^4^ Institute of Physiology, School of Medicine, National Yang Ming Chiao Tung University, Taipei, Taiwan; ^5^ Department of Chinese Pharmaceutical Sciences and Chinese Medicine Resources, China Medical University, Taichung, Taiwan; ^6^ Graduate Institute of Integrated Medicine, College of Chinese Medicine, China Medical University, Taichung, Taiwan; ^7^ Department of Biochemical Science and Technology, National Chiayi University, Chiayi, Taiwan

**Keywords:** *Mesona procumbens* Hemsley, triterpene acid, mesonaic acid, sesquiterpene, mesoeudesmol, anti-inflammatory

## Abstract

*Mesona* *procumbens* Hemsley is a plant conventionally processed to provide popular food materials and herbal medicines in Asia. In this study, six triterpene acids, including five new ones (mesonaic acids D-H, **1**–**5**), and one proximadiol-type sesquiterpene (**7**) were isolated from the methanolic extract of the air-dried *M. procumbens*. Chemical structures of **1**‒**7** were established by spectroscopic methods, especially 2D NMR techniques (^1^H–^1^H COSY, HSQC, HMBC, and NOESY) and HRESIMS. Concerning their biological activities, compounds **1**, **2**, **6**, and **7** were examined manifesting high inhibition toward the pro-inflammatory NO production with EC_50_ values ranging from 12.88 to 21.21 µM, outrunning the positive control quercetin (24.12 µM). The mesoeudesmol B (**7**) identified from *M. procumbens* is the very first example, which exhibited high anti-inflammatory activity diminishing the level of the lipopolysaccharide-induced NO in RAW264.7 macrophage cells, thereby suppressing the secretion of pro-inflammatory cytokines TNF-α and IL-6 and the level of two critical downstream inflammatory mediators iNOS and COX-2.

## 1 Introduction

*Mesona procumbens* Hemsley (Hsian-tsao), an annual herb belonging to the Lamiaceae family, is distributed in the tropical and subtropical regions of South Asia, such as Taiwan, Indonesia, Thailand, Vietnam, and southern China (Feng et al., 2012). This herb is conventionally used alone as a heat-clearing (Qingre) and detoxifying (Jiedu) agent or filled in a prescription of traditional Chinese medicine typically for the treatment of heat-shock, hypertension, diabetes, hepatic disease, and various inflammations, such as joint and muscle pains (Yen and Hung, 2000; Hung and Yen, 2002). Hsian-tsao (also known as grass jelly herb) is preferably consumed as herbal tea, herbal jelly dessert (grass jelly), or dessert soup given its unique smell, refreshing taste, and medicinal benefits from the major component, polysaccharide gum, which is the most attractive target in this edible plant (Lai and Liao, 2002; Lai et al., 2003; Zhuang et al., 2010). Recent studies further revealed that the Hsian-tsao aqueous extract can stop disease progression of liver fibrosis through its apoptotic effects on the activation of hepatic stellate cells (Yeh et al., 2019). The crude polysaccharides from Hsian-tsao demonstrated a wound healing activity in the streptozotocin-induced diabetic mouse model (Fan et al., 2021). Numerous pharmacological properties, such as anti-inflammatory (Huang et al., 2012), antioxidant (Lin et al., 2018), antihypertensive (Yeh et al., 2009), antimutagenic (Yen et al., 2001), DNA damage protection (Yen et al., 2000), liver fibrosis prevention (Shyu et al., 2008), and renal protective activities, from *M. procumbens* extracts have been independently reported from time to time (Yang et al., 2008). In addition, some such chronic inflammations such as cardiovascular, cancer, diabetes, arthritis, pulmonary, and autoimmune diseases reported (Singh et al., 2019) can be improved by natural products for they possess some yet-unknown chemo-preventive activities (Azab et al., 2016; Huang et al., 2021).

This study aimed at investigating untapped anti-inflammatory ingredients from Hsian-tsao rather than the known polysaccharide gum. Seven new chemical entities were identified herein, including five new triterpene acids (two 24-nor-oleanane-type triterpenes **1**‒**2**, one 24-nor-ursane-type triterpene **3**, one ursane-type triterpene acid **4**, and one ursane-type *seco*-triterpene acid **5**), one known 2α,3α,19α-trihydroxy-24-norursa-4 (23),12-dien-28-oic acid (**6**), and one new proximadiol (cryptomeridiol)-type sesquiterpene **7** isolated from the methanolic extract of *M. procumbens*. Having identified their chemical structures, these compounds were subjected to biochemical assay for evaluating their anti-inflammatory capacities against the lipopolysaccharide-induced NO production in RAW264.7 macrophage cells.

## 2 Materials and methods

### 2.1 General experimental procedures

Optical rotations were determined by a JASCO P-2000 polarimeter at 25°C. Infrared (IR) spectra were recorded on a Thermo Scientific Nicolet iS5 FTIR spectrometer. The ECD spectra were measured by a JASCO J-715 spectropolarimeter. High-resolution electrospray ionization mass spectrometry (HRESIMS) data were established by a Thermo Scientific Ultimate 3000 UHPLC System with a Thermo Scientific Q Exactive™ Focus Hybrid Quadrupole-Orbitrap Mass Spectrometer. NMR (nuclear magnetic resonance) spectra, including ^1^H, ^13^C, DEPT, ^1^H–^1^H COSY, HMBC, HSQC, and NOESY, were recorded on Varian Unity Inova 500 MHz (5 mm SWPFG/TRPFG probe) or Varian VNMRS 600 MHz spectrometers (cold probe) and the chemical shifts were referenced by deuterated solvent methanol-*d*_4_. Silica gel 60 (Merck, 70–230 and 230–400 meshes), C_18_ gel (Chromatorex, 40–75 mesh), Diaion HP-20 (Mitsubishi Chemical Co.), and Sephadex LH-20 (GE) were used for column chromatography. Preparative HPLC (high-performance liquid chromatography) was performed using a Shimadzu LC-8A pump and an SPD-10A VP UV detector (210 and 254 nm wavelengths) with a Cosmosil 5C_18_ AR-II column (250 × 20 mm, Nacalai Tesque). TLC (thin-layer chromatography) analyses were conducted on pre-coated silica gel plates (Merck, Kieselgel 60 F_254_, 1 mm) and sprayed with anisaldehyde–sulfuric acid reagent and then heated at 100°C.

### 2.2 Plant material

The whole plants of the air-dried *M. procumbens* Hemsley (8.0 kg) were purchased from Starsci Biotech company in September 2019. A voucher specimen (No. NRICM-20190901) was deposited in the Herbarium of Division of Chinese Materia Medica Development, NRICM, Taipei, Taiwan.

### 2.3 Extraction, isolation, and purification

The air-dried plant of *M. procumbens* Hemsley (8.0 kg) was shredded into 5 mm and extracted with methanol (80 L) at 50°C thrice, and the combined extract was concentrated under reduced pressure removing methanol to obtain the methanolic crude extract. The crude extract (ca. 796.5 g) was dissolved in ddH_2_O and then the aqueous solution was further sequentially partitioned with *n*-hexane and dichloromethane (CH_2_Cl_2_) to obtain the hexane, CH_2_Cl_2_, and aqueous extracts. The CH_2_Cl_2_ extract (ca. 47.6 g) was fractionated by a C_18_ gel flash column (15 × 25 cm) eluting with a 20%, 40%, 60%, 80%, 90%, and 100% MeOH/H_2_O, successively, to yield six fractions (Fr. I‒VI). Fr. IV was separated by chromatography on a flash column (silica gel, 60–230 mesh, 15 × 20 cm) eluting with CH_2_Cl_2_ and acetone (from 5% to 100% acetone) to obtain eight subfractions (Fr. IVA ∼ H). Fr. IVF was separated by preparative HPLC on a Cosmosil 5C_18_ AR-II column (250 × 20 mm, flow rate: 10.0 ml/min) with 60% acetonitrile (ACN) in H_2_O to afford seven subfractions (Fr. IVF-1∼7). Fr. IVF-4 was further purified by HPLC with Cosmosil 5C_18_ AR-II column (flow rate: 10.0 ml/min), eluting with 45% ACN to afford **2** (2.1 mg, *R*_t_: 33.7 min). Using the same HPLC and RP column, eluting with 50% ACN, compound **5** (2.6 mg, *R*_t_: 21.3 min) was purified. Fr. IVE was fractionated by HPLC (Cosmosil 5C_18_ AR-II column, 250 × 20 mm, flow rate: 10.0 ml/min) with 55% ACN to afford eight subfractions (Fr. IVE-1∼8). Fr. IVE-3 was further purified again with 45% ACN (flow rate: 10.0 ml/min) to yield **3** (1.9 mg, *R*_t_: 18.3 min). Fr. IVD was subjected to preparative HPLC with 45% ACN (flow rate: 10.0 ml/min) to yield six subfractions (Fr. IVD-1∼6). Fr. IVD-4 was further repeatedly purified with 40% ACN (flow rate: 10.0 ml/min) to afford compounds **4** (2.3 mg, *R*_t_: 17.9 min) and **7** (4.8 mg, *R*_t_: 26.2 min). Fr. IVC was subjected to HPLC with 45% ACN (flow rate: 10.0 ml/min) to divide into eight subfractions (Fr. IVC-1∼5)_._ Fr. IVC-2 was separated by a Cosmosil 5C_18_ AR-II column (flow rate: 10.0 ml/min) to afford **1** (1.6 mg, *R*_t_: 31.2 min). Fr. IVC-4 was also purified by HPLC using the same RP column with 40% ACN (flow rate: 10.0 ml/min) to afford **6** (2.5 mg, *R*_t_: 49.9 min).

### 2.4 Spectroscopic data

#### 2.4.1 Mesonaic acid D (**1**)

White amorphous powder; [*α*] +12.9 (*c* 0.5, MeOH); IR (KBr) ν_max_ 3402, 2927, 1694, 1437, 1316, 1017 cm^−1^; ^1^H- (600 MHz) and ^13^C- (150 MHz) NMR spectroscopic data (methanol-*d*_4_) are shown in Tables 1, 2, respectively; HRESIMS *m/z* 495.3097 [M + Na]^+^ (calcd. for C_29_H_44_O_5_Na, 495.3081).

**TABLE 1:** ^1^H-NMR spectroscopic data of **1**‒**6** in methanol-*d*_4_.

No	1^a^	2^a^	3^a^	4^a^	5^b^	6^a^
1	1.35 m	1.23 dd (12.0, 12.6)	2.14 brd (16.2)	1.34 m	2.39 d (18.0)	1.38 m
	1.70 dd (4.8, 12.0)	1.67 dd (6.6, 12.6)	2.55 d (16.2)	1.53 m	2.58 d (18.0)	1.71 m
2	3.65 ddd (3.6, 4.8, 12.0)	3.68 ddd (3.6, 4.8, 11.4)	-	3.89 ddd (3.0, 4.8, 12.0)	-	3.67 ddd (3.6, 4.8, 12.0)
3	4.12 d (3.6)	4.11 d (3.6)	-	3.73 brd (3.0)	-	4.13 d (3.6)
5	2.17 dd (4.2, 9.6)	2.22 br d (13.2)	2.41 brd (12.0)	1.91 m	2.53 dd (3.0, 12.5)	2.18 m
6	1.46 m (2H)	1.48 m	1.47 m	1.43 m (2H)	1.53 m	1.46 m (2H)
		1.62 m	1.88 m		1.35 m	
7	1.34 m	1.32 m	1.43 m	1.23 m	1.35 m	1.34 m
	1.58 m	1.98 td (3.0, 13.2)	1.71 td (4.2, 12.6)	1.74 m	1.66 m	1.63 m
9	1.82 m	1.21 m	2.05 m	1.94 m	2.76 dd (6.5, 11.5)	1.93 m
11	1.94 m	1.32 m	2.03 m (2H)	1.96 m	1.99 m	1.98 m
	2.02 m	1.55 m		2.02 m	2.08 m	2.14 m
12	5.28 t (3.6)	4.00 brdd (2.4, 5.4)	5.33 t (3.6)	5.28 t (3.6)	5.31 t (4.0)	5.30 t (3.0)
15	1.10 m	5.02 d (5.4)	1.06 m	0.99 m	1.10 m	1.01 brd (13.8)
	1.80 m		1.81 m	1.79 td (5.4, 13.8)	1.89 m	1.83 td (4.8, 13.8)
16	1.61 m	1.64 m	1.83 m	1.50 m	1.90 m	1.52 m
	2.02 m	2.13 d (12.6)	2.56 td (4.2, 12.6)	2.57 td (4.8, 13.2)	2.28 m	2.58 td (4.8, 13.2)
18	2.88 dd (4.8, 14.4)	1.88 dd (4.2, 12.6)	2.69 brs	2.49 brs	2.49 brs	2.50 brs
19	1.08 m	1.18 m	-	-	-	-
	1.80 m	1.82 dd (4.8, 13.8)				
20	-	-	1.57 m	1.33 m	1.50 m	1.34 m
21	1.15 m	1.09 td (4.2, 13.2)	3.90 dd (3.0, 6.0)	1.22 m	1.45 dd (4.5, 12.5)	1.22 m
	1.48 m	1.30 m		1.71 m	1.85 dd (12.0, 12.5)	1.71 m
22	1.57 m	1.17 m	1.81 m	1.60 td (4.8, 13.8)	3.71 dd (4.5, 12.0)	1.61 m
	1.67 m	2.09 td (4.2, 15.0)	2.10 dd (3.0, 14.4)	1.71 m		1.72 m
23	4.67 brs	4.99 brs	1.84 d (1.8)	1.19 s	1.29 s	4.68 brs
	5.00 brs	4.85 brs				5.01 brs
24	-	-	*-*	-	1.28 s	-
25	0.76 s	0.66 s	0.93 s	1.03 s	1.08 s	0.78 s
26	0.86 s	1.17 s	0.88 s	0.79 s	0.85 s	0.84 s
27	1.21 s	0.74 d (7.2)	1.36 s	1.38 s	1.40 s	1.36 s
28		1.19 m				
29	0.92 s	0.94 s	1.16 s	1.18 s	1.17 s	1.19 s
30	3.18 brs (2H)	0.98 s	1.16 d (6.6)	0.92 d (6.6)	0.98 d (6.5)	0.92 d (6.6)

a ^1^H-NMR data were recorded on 600 MHz.

b ^1^H-NMR data were recorded on 500 MHz.

**TABLE 2:** ^13^C-NMR spectroscopic data of **1**‒**6** in methanol-*d*_4_.

No	1^a^		2^a^		3^a^		4^a^		5^b^		6^a^	
1	42.0	CH_2_	42.1	CH_2_	51.8	CH_2_	40.1	CH_2_	41.9	CH_2_	42.0	CH_2_
2	68.7	CH	68.8	CH	194.2	qC	65.4	CH	174.2	qC	68.8	CH
3	75.6	CH	75.4	CH	143.9	qC	75.4	CH	182.4	qC	75.7	CH
4	151.0	qC	150.5	qC	131.6	qC	51.8	qC	46.1	qC	151.1	qC
5	44.4	CH	45.1	CH	48.5	CH	43.4	CH	48.4	CH	44.5	CH
6	20.0	CH_2_	19.9	CH_2_	20.5	CH_2_	20.5	CH_2_	21.2	CH_2_	20.2	CH_2_
7	31.1	CH_2_	37.0	CH_2_	32.0	CH_2_	32.3	CH_2_	32.0	CH_2_	31.3	CH_2_
8	39.3	qC	39.5	qC	39.1	qC	39.9	qC	39.7	qC	39.8	qC
9	45.0	CH	43.2	CH	43.4	CH	47.1	CH	39.2	CH	44.5	CH
10	37.3	qC	36.9	qC	40.9	qC	37.5	qC	40.2	qC	37.3	qC
11	23.9	CH_2_	28.9	CH_2_	23.4	CH_2_	23.2	CH_2_	23.6	CH_2_	24.1	CH_2_
12	122.4	CH	73.0	CH	128.0	CH	127.8	CH	128.4	CH	128.1	CH
13	144.1	qC	33.8	qC	138.2	qC	138.8	qC	138.0	qC	138.9	qC
14	41.8	qC	36.1	qC	41.6	qC	41.4	qC	42.4	qC	41.5	qC
15	27.3	CH_2_	78.0	CH	28.4	CH_2_	28.2	CH_2_	27.7	CH_2_	28.1	CH_2_
16	22.6	CH_2_	33.0	CH_2_	27.6	CH_2_	25.2	CH_2_	18.0	CH_2_	25.2	CH_2_
17	46.5	qC	45.2	qC	47.2	qC	47.6	qC	53.5	qC	47.6	qC
18	40.7	CH	39.2	CH	54.1	CH	53.7	CH	54.5	CH	53.8	CH
19	39.9	CH_2_	41.0	CH_2_	75.2	qC	72.1	qC	71.8	qC	72.1	qC
20	35.4	qC	29.5	qC	41.8	CH	41.7	qC	39.9	qC	41.7	qC
21	27.9	CH_2_	34.2	CH_2_	73.1	CH	25.9	CH_2_	34.3	CH_2_	25.8	CH_2_
22	31.7	CH_2_	27.7	CH_2_	43.4	CH_2_	37.6	CH_2_	74.2	CH	37.6	CH_2_
23	109.4	CH_2_	109.0	CH_2_	11.9	CH_3_	16.2	CH_3_	26.3	CH_3_	109.3	CH_2_
24							178.5	qC	23.5	CH_3_		
25	13.0	CH_3_	13.5	CH_3_	13.0	CH_3_	15.9	CH_3_	18.2	CH_3_	13.0	CH_3_
26	16.4	CH_3_	20.0	CH_3_	16.3	CH_3_	16.3	CH_3_	16.0	CH_3_	16.2	CH_3_
27	25.1	CH_3_	15.7	CH_2_	22.5	CH_3_	23.6	CH_3_	23.1	CH_3_	23.6	CH_3_
28	180.3	qC	182.3	qC	180.1	qC	180.8	qC	179.0	qC	180.9	qC
29	18.1	CH_3_	32.0	CH_3_	25.1	CH_3_	25.6	CH_3_	25.3	CH_3_	25.6	CH_3_
30	73.0	CH_2_	22.7	CH_3_	12.5	CH_3_	15.2	CH_3_	15.0	CH_3_	15.2	CH_3_

a ^13^C- and DEPT NMR data were recorded on 150 MHz.

b ^13^C- and DEPT NMR data were recorded on 125 MHz.

#### 2.4.2 Mesonaic acid E (**2**)

White amorphous powder; [*α*] +19.7 (*c* 0.5, MeOH); IR (KBr) ν_max_ 3399, 2930, 1744, 1390, 1245, 1012 cm^−1^; ^1^H- (600 MHz) and ^13^C- (150 MHz) NMR spectroscopic data (methanol-*d*_*4*_) are shown in Tables 1, 2, respectively; HRESIMS *m/z* 469.2965 [M ‒ H]^‒^ (calcd. for C_29_H_41_O_5_, 469.2949).

#### 2.4.3 Mesonaic acid F (**3**)

White amorphous powder; [*α*] +25.1 (*c* 0.5, MeOH); IR (KBr) ν_max_ 3369, 2936, 1712, 1630, 1385, 1170, 1017 cm^−1^; ^1^H- (600 MHz) and ^13^C- (150 MHz) NMR spectroscopic data (methanol-*d*_4_) are shown in Tables 1, 2, respectively; HRESIMS *m/z* 485.2919 [M ‒ H]^‒^ (calcd. for C_29_H_41_O_6_, 485.2898).

#### 2.4.4 Mesonaic acid G (**4**)

White amorphous powder; [*α*] +23.4 (*c* 0.5, MeOH); IR (KBr) ν_max_ 3402, 2927, 1687, 1235, 1022 cm^−1^; ^1^H- (600 MHz) and ^13^C- (150 MHz) NMR spectroscopic data (methanol-*d*_4_) are shown in Tables 1, 2, respectively; HRESIMS *m/z* 517.3176 [M ‒ H]^‒^ (calcd. for C_30_H_45_O_7_, 517.3160).

#### 2.4.5 Mesonaic acid H (**5**)

White amorphous powder; [*α*] +27.3 (*c* 0.5, MeOH); IR (KBr) ν_max_ 3414, 2952, 1707, 1462, 1163, 1027 cm^−1^; ^1^H- (500 MHz) and ^13^C- (125 MHz) NMR spectroscopic data (methanol-*d*_4_) are shown in Tables 1, 2, respectively; HRESIMS *m/z* 533.3124 [M ‒ H]^‒^ (calcd. for C_30_H_45_O_8_, 533.3109).

#### 2.4.6 2α,3α,19α-Trihydroxy-24-norursa-4 (23),12-dien-28-oic acid (**6**)

White amorphous powder; [*α*] +21.8 (*c* 0.5, MeOH); IR (KBr) ν_max_ 3470, 2947, 1697, 1459, 1247, 1039 cm^−1^; ^1^H- (600 MHz) and ^13^C- (150 MHz) NMR spectroscopic data (methanol-*d*_4_) are shown in Tables 1, 2, respectively; HRESIMS *m/z* 495.3072 [M + Na]^+^ (calcd. for C_29_H_44_O_5_Na, 495.3081).

#### 2.4.7 Mesoeudesmol B (**7**)

Colorless oil; [*α*] +9.1 (*c* 0.5, MeOH); IR (KBr) ν_max_ 3389, 2932, 1716, 1455, 1388, 1279, 1116, 1025 cm^−1^; UV λ_max_ (MeOH) (log ε) 273 (2.98), 229 (3.82) nm; ^1^H- (600 MHz) and ^13^C- (150 MHz) NMR spectroscopic data (methanol-*d*_4_) are shown in Table 3; HRESIMS *m/z* 399.2148 [M + Na]^+^ (calcd. for C_22_H_32_O_5_Na, 399.2142).

**TABLE 3:** ^1^H- and ^13^C-NMR spectroscopic data of **7** in methanol-*d*_4_.

No	^1^H NMR (600 MHz)	^13^C NMR (150 MHz)		No	^1^H NMR (600 MHz)	^13^C NMR (150 MHz)	
1	1.31 m	46.0	CH_2_	12	3.46 s (2H)	67.7	CH_2_
	1.88 m						
2	5.18 ddd (4.2, 7.8, 12.0)	69.3	CH	13	1.12 s	19.7	CH_3_
3	1.62 m	47.7	CH_2_	14	1.19 brs	22.0	CH_3_
	2.21 ddd (2.4, 4.2, 12.0)						
4	-	72.0	qC	15	1.02 s	18.5	CH_3_
5	1.34 m	53.8	CH	1′	-	166.0	qC
6	1.45 m	20.7	CH_2_	2′	-	130.4	qC
	1.65 m						
7	1.58 m	44.5	CH	3′	7.98 dd (1.8, 8.0)	129.0	CH
8	1.12 m	21.1	CH_2_	4′	7.45 dd (7.2, 8.0)	128.1	CH
	1.88 m						
9	1.27 m	44.3	CH_2_	5′	7.58 m	132.7	CH
	1.54 m						
10	-	33.9	qC	6′	7.45 dd (7.2, 8.0)	128.1	CH
11	-	74.0	qC	7′	7.98 dd (1.8, 8.0)	129.0	CH

### 2.5 Dimolybdenum tetraacetate [Mo_2_(OAc)_4_]-modified circular dichroism analysis

The determination of the absolute configuration of cyclic and acyclic *vic*-diols was achieved by employing a transition metal chelate reagent, dimolybdenum tetraacetate [Mo_2_(OAc)_4_]. Compound **7** was directly dissolved in a solution of Mo_2_(OAc)_4_ complex in DMSO in a molar ratio of Mo_2_(OAc)_4_/compound of about 1:0.3–1:0.7, and the mixture was subsequently measured for the induced CD spectra without the preparation and isolation of the complexes.

### 2.6 Cell culture and viability assay

The RAW264.7 mouse macrophages were purchased from the Food Industry Research and Development Institute (Hsinchu, Taiwan). Cells were cultured in DMEM supplemented with 10% heat-inactivated FBS in a 5% CO_2_ humidified incubator at 37°C. For viability assay, RAW264.7 cells were pretreated with various concentrations of the isolated compounds (0, 5, 10, and 20 μM) 1 h prior to LPS (1 μg/ml) stimulation. After 24 h treatment, cell viability was determined by Cell Counting Kit-8 (Dojindo, Rockville, MD, United States) according to the manufacturer’s instructions.

### 2.7 NO releasing inhibition assay

Griess reagent (1% sulfanilamide in 5% phosphoric acid and 0.1% naphthylethylenediamine dihydrochloride in water) was used to determine NO production. RAW264.7 cells were pretreated in the same manner described in cell viability assay. 100 µL of supernatant of each pretreated cell solution was transferred to a new microtiter plate, and each supernatant was mixed with 100 µL of Griess reagent. The microtiter plate was left at room temperature for 10 min for color development, and each solution was measured by a microplate reader at UV 540 nm. All experiments were performed in triplicate.

### 2.8 Western blot analysis

RAW264.7 cells were pretreated with compound **7** (0, 5, 10, or 20 μM) or quercetin (25 μM, Sigma-Aldrich) for 1 h and then stimulated with LPS for an additional 24 h. The cells were lysed in radioimmunoprecipitation assay (RIPA) lysis buffer and protein concentration was determined using the Bradford assay (Bio-Rad Laboratories, Munich, Germany). The protein lysates were separated by SDS-PAGE and then transferred to a PVDF membrane. The blot was blocked with TBS containing 5% nonfat milk for 1 h at room temperature and then incubated with primary antibodies to iNOS, COX-2 or β-actin at 4°C overnight. The blots were washed three times with 0.1% TBST (0.1% Tween 20 in TBS) and then incubated with a peroxidase-conjugated secondary antibody for 1 h at room temperature. After washing, the protein bands were detected using an ECL reagent and X-ray film.

### 2.9 Determination of IL-6 and TNF-α levels

The measurement of the IL-6 and TNF-α levels was performed using commercial ELISA kits (RAB0308-1KT and RAB0477-1KT (Sigma Chemical Co., St. Louis, MO, United States), respectively) according to the manufacturer’s instructions. Briefly, samples and standards were added to antibody-coated 96 wells and were incubated for 2.5 h at room temperature. After incubation, wells were washed with wash buffer four times, and then detection antibody was added and incubated for another 1 h at room temperature. After washing, 3,3′,5,5′-tetramethylbenzidine (TMB) substrate was added and incubated in the dark for 30 min at room temperature followed by adding stop solution and reading absorbance at 450 nm immediately.

### 2.10 Statistical analysis

Statistical analyses were performed using SPSS (SPSS, Chicago, IL, United States). Data are expressed as the mean ± standard deviations. Statistical significance was determined using one-way ANOVA analysis followed by Tukey’s test. *p*-values < 0.05 were considered statistically significant.

## 3 Results and discussion

**FIGURE 1:**
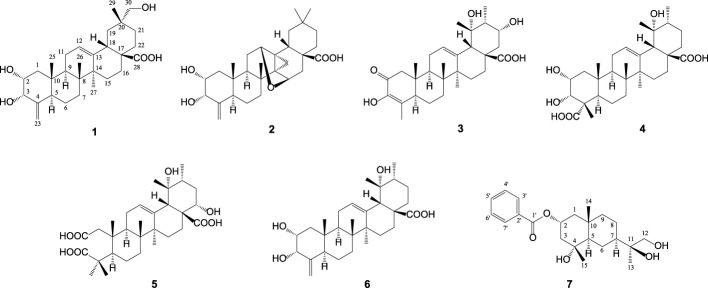
Chemical structures of compounds **1**‒**7** isolated from *M. procumbens*.

The methanolic extract of *M. procumbens* was dried and resuspended in H_2_O; this aqueous solution was extracted by *n*-hexane and CH_2_Cl_2_ to give rise to two organic layers. The CH_2_Cl_2_ portion was subjected to chromatography by a flash column and preparative RP-HPLC to afford five new and one known triterpene acids (**1**–**6**), together with one brand new sesquiterpene (**7**) (Figure 1). All pure components (**1**–**7**) were then evaluated for their anti-inflammatory activities using an *in vitro* LPS-stimulated murine macrophage model.

### 3.1 Structural elucidation of the isolated compounds

**FIGURE 2:**
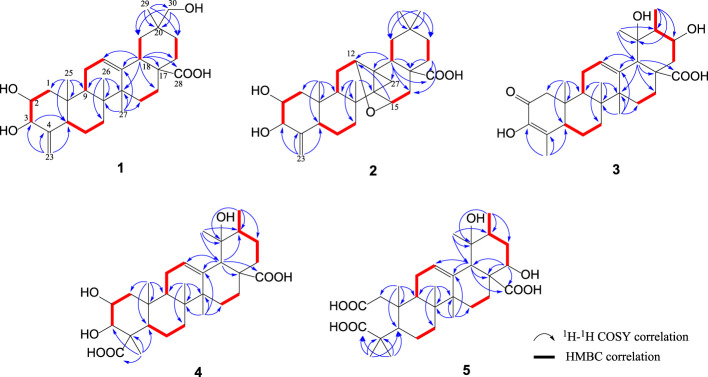
^1^H–^1^H COSY and key HMBC correlations of triterpene acids **1**‒**5**.

Compound **1** ([*α*] +12.9, *c* = 0.5, MeOH), a colorless amorphous powder, has a molecular formula of C_29_H_44_O_5_ with 8 degrees of unsaturation (DOU) deduced from the sodiated HRESIMS pseudo-ion at *m/z* 495.3097 [M + Na]^+^ (calcd. for C_29_H_44_O_5_Na, 495.3081). In ^1^H-NMR spectrum (Table 1), four tertiary methyl signals (*δ*_H_ 0.76, 0.86, 0.92, and 1.21 s), one olefinic methylene signal (*δ*_H_ 4.67 brs and 5.00 brs), and one olefinic methine signal (*δ*_H_ 5.28 t, *J* = 3.6 Hz) were observed. Based on ^13^C-NMR and DEPTs spectra (Table 2), the 29 carbons can be categorized into 4 methyls, 11 methylenes (one oxymethylene at *δ*_C_ 73.0 and one olefinic methylene at *δ*_C_ 109.4), 6 methines (two oxymethines at *δ*_C_ 68.7 and 75.6 as well as one olefinic methine at *δ*_C_ 122.4), and 8 quaternary carbons (two sp^2^ quaternary carbons at *δ*_C_ 144.1 and 151.0 and one carboxylic acid group at *δ*_C_ 180.3). The above two olefins and one carboxyl group accounted for three DOU, and the remaining five constrained **1** to a pentacyclic structure. The assignments of ^1^H- and ^13^C-NMR spectroscopic data were completed by a combination of HSQC, ^1^H–^1^H COSY, and HMBC experiments. By comparison of carbon signals of **1** with that of oleanolic acid along with the analysis of the COSY and HMBC correlations of **1** altogether determined the carbon skeleton of **1**, an oleanane-type nortriterpenoid (Kashiwada et al., 1998). In Figure 2, the COSY correlations of H-2 (*δ*_H_ 3.65 ddd, *J* = 3.6, 4.8, 12.0 Hz)/H-3 (*δ*_H_ 4.12 d, *J* = 3.6 Hz) confirmed that the attachments of these two hydroxyls are at C-2 and C-3, whereas the HMBC correlations from H_2_-30 (*δ*_H_ 3.18 brs) to C-19 (*δ*_C_ 39.9), C-20 (*δ*_C_ 35.4), and C-21 (*δ*_C_ 27.9) revealed a primary alcohol at C-30. Furthermore, an *exo*-4 (23)-double bound was verified according to the HMBC correlations of olefinic methylene H_2_-23 to C-3 (*δ*_C_ 75.6), C-4 (*δ*_C_ 151.0), and C-5 (*δ*_C_ 44.4); the tri-substituted Δ^12^ double bond was confirmed by the COSY correlations of H-9 (*δ*_H_ 1.82 m)/H_2_-11 (*δ*_H_ 1.94 and 2.02 m)/H-12 (*δ*_H_ 5.28 t, *J* = 3.6 Hz) and by the HMBC correlations from H-18 (*δ*_H_ 2.88 dd, *J* = 4.8 and 14.4 Hz) to C-12 (*δ*_C_ 122.4), C-13 (*δ*_C_ 144.1), and C-14 (*δ*_C_ 41.8). The spectroscopic evidence altogether indicated that **1** is a new 24-noroleanane-type triterpene acid.

**FIGURE 3:**
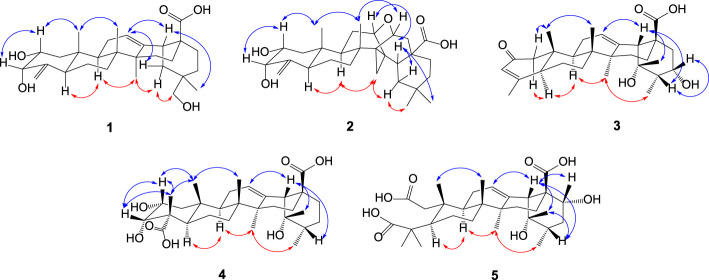
Main NOESY correlations of triterpene acids **1**‒**5**.

The pentacyclic moiety of oleanane-type triterpenoids is rigid, showing the configurations with those of regular oleanane-type triterpenoids based on NOESY experiment, which provides clear assignments. The NOESY correlations of H-5/H-9, H-9/CH_3_-27, CH_3_-27/H_α_-19, and H_α_-19/H_2_-30 indicated α-orientations, whereas those of CH_3_-25, CH_3_-26, H-18, and CH_3_-29 presented β-directions with regard to H-18/CH_3_-25, CH_3_-25/CH_3_-26, and H-18/CH_3_-29 (Figure 3). Meanwhile, the NOESY correlations of H-2/H-3 and H-2/CH_3_-25 and the small proton constant of H-3 (^3^ *J*_H-2,H-3_ = 3.6 Hz) agreed with the α-oriented OH-2 and OH-3 within a *cis*-relationship. As a result, the chemical structure of mesonaic acid D (**1**) was established as 2α,3α,30-trihydroxy-24-norolean-4 (23),12-dien-28-oic acid.

Mesonaic acid E (**2**) was white amorphous powders with [*α*] +19.7 (*c* 0.5, MeOH). The molecular formula C_29_H_42_O_5_ on par with 9 DOU was deduced based on a quasimolecular ion at *m/z* 469.2965 [M ‒ H]^‒^ (calcd. for C_29_H_41_O_5_, 469.2949) in the HRESIMS experiment. The characteristic ^1^H- and ^13^C-NMR signals (Tables 1, 2), four tertiary methyls (*δ*_H_ 0.66 s/*δ*_C_ 13.5; *δ*_H_ 0.94 s/*δ*_C_ 32.0; *δ*_H_ 0.98 s/*δ*_C_ 22.7; *δ*_H_ 1.17 s/*δ*_C_ 20.0), two oxymethines C-2 (*δ*_H_ 3.68 ddd, *J* = 3.6, 4.8, 11.4 Hz/*δ*_C_ 68.8) and C-3 (*δ*_H_ 4.11 d, *J* = 3.6 Hz/*δ*_C_ 75.4), one exocyclic methylene involving C-4 (*δ*_C_ 150.5) and C-23 (*δ*_H_ 4.85 brs and 4.99 brs/*δ*_C_ 109.0), and one COOH-28 (*δ*_C_ 180.3) suggested that compound **2** is a derivative of 24-nor-oleanane triterpene acid possessing the same 2,3-diol ring A as **1**. Deduction of DOU from the 4 (23)-double bond, COOH-28, and the pentacyclic ring moiety, there are two DOU unassigned. After a detailed comparison of ^1^H-, ^13^C-NMR, and DEPTs spectra, we found that a tri-substituted Δ^12^ double bond and the primary alcohol of C-30 disappeared, while two oxymethines (*δ*_H_ 4.00 dd, *J* = 2.4 and 5.4 Hz/*δ*_C_ 73.0 and *δ*_H_ 5.02 d, *J* = 5.4 Hz/*δ*_C_ 78.0), one aliphatic methylene (*δ*_H_ 0.74 d, *J* = 7.2 Hz/*δ*_C_ 15.7), and one aliphatic quaternary carbon (*δ*_C_ 33.8) emerged. By a combination of the COSY correlations of H-9/H_2_-11/H-12 (indicating that olefinic methine C-12 converts to an oxymethine) and the HMBC correlations from another oxymethine H-15 to C-12, one DOU was contributed to an oxygen-bridge between C-12 and C-15. The remaining DOU is resulted from a cyclopropane unit assembled by one aliphatic methylene C-27 and two aliphatic quaternary carbons C-13 and C-14, which were confirmed by the HMBC correlations (Figure 2) of H-18 (*δ*_H_ 1.88 dd, *J* = 4.2 and 12.6 Hz)/C-12, C-13, C-17 (*δ*_C_ 45.2), C-27, and COOH-28 and H_3_-26 (*δ*_H_ 1.17 s)/C-7 (*δ*_C_ 37.0), C-8 (*δ*_C_ 39.5), C-9 (*δ*_C_ 43.2), and C-14 (*δ*_C_ 36.1).

In terms of stereochemistry, the chiral centers at ring junctions of **2** are carried on as those at compound **1**; The α-side of the cyclopropane unit (C-13, C-14, and C-27) which formed via a si-attack by α-oriented CH_3_-27 were supported by the NOESY correlations (H-9/H_2_-27, H_2_-27/H_α_-19, and H_α_-19/CH_3_-30) (Figure 3). The 2α,3α-diol is also confirmed by the NOESY correlations of H-2/H-3 and H-2/CH_3_-25 as well as the small proton constant of H-3 (*J* = 3.6 Hz). The configurations of the oxygen bridge heads, C-12 and C-15, were determined to be at the β-side based on the NOESY correlations of H-12/H-18 and H-15/CH_3_-26. Thereby, compound **2** was clearly elucidated as 2α,3α-dihydroxy-12 (15)-epoxy-13α,27-cycloolean-4 (23)-en-28-oic acid.

Mesonaic acid F (**3**) was obtained as a white powder with specific rotation [*α*] +25.1 (*c* 0.5, MeOH). Its IR spectrum showed the presence of a hydroxyl group (3369 cm^−1^), a carboxyl group (1712 cm^−1^), a conjugated carbonyl group (1630 cm^−1^), and an olefinic group (1385 cm^−1^). The molecular formula of **3** was determined to be C_29_H_42_O_6_ (9 DOU) by the given HRESIMS ion at *m/z* 485.2919 [M ‒ H]^‒^ (calcd. 485.2898 for C_29_H_41_O_6_). The 29 carbons (Table 2) can be separated into 6 methyls (*δ*_C_ 11.9, 12.5, 13.0, 16.3, 22.5, and 25.1), 7 methylenes (*δ*_C_ 20.5, 23.4, 27.6, 28.4, 32.0, 43.4, and 51.8), 6 methines (*δ*_C_ 41.8, 43.4, 48.5, 54.1, 73.1, and 128.0), and 10 quaternary carbons (*δ*_C_ 39.1, 40.9, 41.6, 47.2, 75.2, 131.6, 138.2, 143.9, 180.1, and 194.2) by ^13^C-NMR and DEPTs spectra of **3**. The protons of five methyls [*δ*_H_ 0.88, 0.93, 1.36 s, 1.16 d, (*J* = 6.6 Hz), and 1.84 d (*J* = 1.8 Hz)], one oxymethine (*δ*_H_ 3.90 dd, *J* = 3.0 and 6.0 Hz), and one olefinic methine (*δ*_H_ 5.33 t, *J* = 3.6 Hz) can be clearly observed in ^1^H-NMR spectrum (Table 1). Combining the ^1^H-, ^13^C-NMR, and HSQC experiments, compound **3** was figured out to be a pentacyclic triterpene acid containing one conjugated carbonyl group, one tri-substituted double bond, and one carboxylic acid, reconciling these functional groups and pentacyclic fused ring system with the 9 DOU. The COSY correlations of H-9/H_2_-11/H-12 and the HMBC correlations from a junction proton H-18 (*δ*_H_ 2.69 brs) to C-12, C-13, C-14, C-17 and C-28 together made a tri-substituted double bond at the Δ^12^ position clear; the ^1^H–^1^H COSY correlations of CH_3_-30/H-20/H-21/H_2_-22 alongside the HMBC correlations of CH_3_-29/C-18, C-19, C-20 illustrated that **3** is a derivative of ursane-type triterpenoids. The most difficult part is that the α-hydroxy-β-methyl substitution in the α,β-unsaturated conjugated carbonyl part in ring A is deduced by the HMBC correlations of CH_3_-23 to C-3, C-4, C-5 and CH_3_-25 to C-1, C-9, C-10, revealing the ursane triterpenoid without the C-24 carbon in **3** (Figure 2). The chiral configurations of **3** were built up by the NOESY correlations (Figure 3) and comparison with a pomolic acid (Kashiwada et al., 1998). As a result, **3** is identified as 3,19α,21α-trihydroxy-2-oxo-24-norurs-3,12-dien-28-oic acid.

Compound **4**, [*α*] +23.4 (*c* 0.5, MeOH), was purified as white amorphous powders. The molecular formula C_30_H_46_O_7_ (8 DOU) was determined by the HRESIMS molecular ion at *m/z* 517.3176 [M-H]^‒^ (calcd. for C_30_H_45_O_7_, 517.3160). The ^1^H-, ^13^C-NMR (Tables 1, 2), and HSQC spectra of **4** as a whole revealed the presence of six methyls (*δ*_H_ 0.79 s/*δ*_C_ 13.6; *δ*_H_ 0.92 d, *J* = 6.6 Hz/*δ*_C_ 15.2; *δ*_H_ 1.03 s/*δ*_C_ 15.9; *δ*_H_ 1.18 s/*δ*_C_ 25.6; *δ*_H_ 1.19 s/*δ*_C_ 16.2; and *δ*_H_ 1.38 s/*δ*_C_ 23.6), one tri-substituted olefinic double bond (*δ*_H_ 5.28 t, *J* = 3.6 Hz/*δ*_C_ 127.8 and *δ*_C_ 138.8), and two carboxyl groups (*δ*_C_ 178.5 and 180.8), the characteristics of urs-12-ene or olean-12-ene derivatives. In addition, two secondary hydroxyl groups at the C-2 and C-3 positions were amended for **4** because of the (C)−CH_2_−CHOH−CHOH−(C) structural moiety (*δ*_H_ 1.34 and 1.53 m; 3.89 ddd, *J* = 3.0, 4.8, and 12.0 Hz; *δ*_H_ 3.73 brd, *J* = 3.0 Hz) established by COSY spectrum. The HMBC spectrum of **4** confirmed the above deductions. Furthermore, the two carboxyl groups were assigned at C-24 (*δ*_C_ 180.8) and C-28 (*δ*_C_ 178.5) according to the HMBC correlations from H-18 (*δ*_H_ 2.49 brs) toC-17 (*δ*_C_ 47.6) and carboxyl carbon C-28 (*δ*_C_ 180.8), from CH_3_-23 to two oxymethine carbons [C-3 (*δ*_C_ 75.4) and C-4 (*δ*_C_ 51.8)], carboxyl carbon C-24 (*δ*_C_ 178.5), respectively. The HMBC correlation from CH_3_-29 to oxygen-bearing quaternary carbon C-19 (*δ*_C_ 72.1) put forward that the tertiary hydroxyl group is at C-19 (Figure 2). The configuration of **4** was established by the NOESY correlations (Figure 3) and the coupling constant interpretations for the relevant protons. The 2α,3α-configurations of the two secondary hydroxyl groups of **4** were ascertained by the NOESY correlations of H-2/H-3, H-2/CH_3_-25, H-3/CH_3_-23, CH_3_-23/CH_3_-25, and the small proton coupling constant of H-3 (*J* = 3.6 Hz) agreed with their directions. These elucidations leaded compound **4** to 2α,3α,19α-trihydroxyurs-12-ene-24,28-dioic acid, a triterpene acid monomer named mesonaic acid G, the first of its kind isolated from *M. procumbens* (it was reported to be a unit in the dimeric triterpene glucoside sanguidioside A) (Liu et al., 2004).

Mesonaic acid H (**5**) was obtained as white powders ([*α*] +27.3 (*c* 0.5, MeOH) with a molecular formula C_30_H_46_O_8_ (8 DOU) determined by the HRESIMS ion at *m/z* 533.3124 [M ‒ H]^‒^ (calcd. for C_30_H_45_O_8_, 533.3109). Compound **5** was thought to be a urs-12-ene derivative where one methyl doublet (*δ*_H_ 0.98 d, *J* = 6.5 Hz), seven methyls, one tri-substituted olefinic double bond (*δ*_H_ 5.31 t, *J* = 4.0 Hz/*δ*_C_ 128.4 and *δ*_C_ 138.0), and three carboxyl groups (*δ*_C_ 174.2, 179.0 and 182.4) were deduced by ^1^H- and ^13^C-NMR spectra (Tables 1, 2). Except for one secondary hydroxyl group and two carboxyl groups, the overall similarity concerning the chemical shifts of remaining carbons made the chemical skeleton of ursane-type triterpene acid with seven unmodified methyls. Based on HMBC correlations (Figure 2), the secondary OH group was assigned at C-22 by the resonances of H-22 (*δ*_H_ 3.71 dd, *J* = 4.5 and 12.0 Hz)/C-17 (*δ*_C_ 53.5) and carboxyl C-28 (*δ*_C_ 179.0). The HMBC correlations of both CH_3_-23 (*δ*_H_ 1.29 s) and CH_3_-24 (*δ*_H_ 1.28 s) to carboxyl C-3 (*δ*_C_ 182.4), C-4 (*δ*_C_ 46.1), and C-5 (*δ*_C_ 48.4) pointed out that there are two carboxyl groups one at C-3 and the other at C-2. It means that the C‒C bond between C-2 and C-3 is broken likely by an oxidative cleavage, and compound **5** has a *seco*-ring A. The stereochemistry of **5** is identical to regular ursane triterpene acids in whichα-orientation of OH-22 is supported by the NOESY correlations as shown in Figure 3. Added together, compound **5** was determined to be 19α,22α-dihydroxy-2,3-secours-12-ene-28-oic acid.

Compound **6**, [*α*] +21.8 (*c* 0.5, MeOH), was isolated as white amorphous powders and had a molecular formula of C_29_H_44_O_5_ determined by sodiated quasimolecular ion at *m/z* 495.3072 [M + Na]^+^ (calcd. for C_29_H_44_O_5_Na, 495.3081) in the HRESIMS experiment. The IR, ^1^H, and ^13^C-NMR spectroscopic data are very similar to compound **4**. Detailed analysis of their data, compound **6** possesses an exocyclic double bond (*δ*_H_ 4.68 brs, 5.01 brs; *δ*_C_ 151.1, 109.3), which replaced a carboxyl group and a singlet methyl in **4**. The complete chemical structure and NMR assignment (Tables 1, 2) were further established and confirmed by 1D and 2D NMR to be identified as 2α,3α,19α-trihydroxy-24-norursa-4 (23),12-dien-28-oic acid (Jang et al., 2005).

**FIGURE 4:**
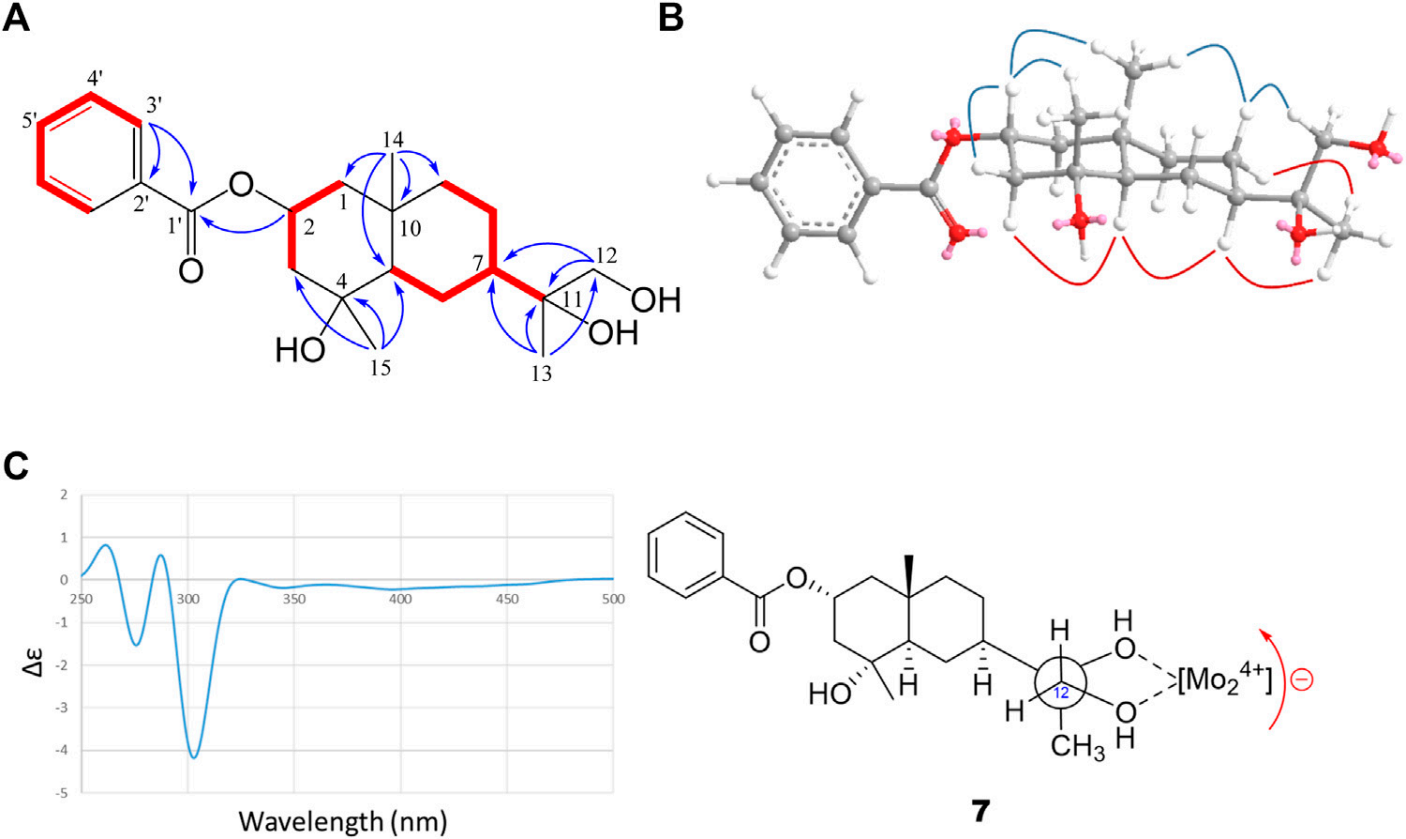
2D correlations and CD spectrum of **7**. **(A)** Key HMBC and ^1^H–^1^H COSY correlations. **(B)** Main NOESY correlations. **(C)** CD spectrum of **7** with Mo_2_(OAc)_4_ in DMSO with the inherent CDs subtracted.

Compound **7** was collected as an oil sample ([*α*] +9.1, *c* 0.5 in MeOH) with a molecular formula C_22_H_32_O_5_ (on par with 7 DOU) deduced from the HRESIMS ion at *m/z* 399.2148 [M + Na]^+^ (calcd. for C_22_H_32_O_5_Na, 399.2142). According to ^13^C-NMR and DEPT spectra (Table 3), the 22 carbons can be divided into three methyls, six methylenes (one oxymethylene at *δ*_C_ 67.7), eight methines [including one oxymethine at *δ*_C_ 69.3 and five sp^2^ methines at *δ*_C_ 128.1 (2), 129.0 (2), and 132.7], and five quaternary carbons (two oxygenated ones at *δ*_C_ 72.0 and 74.0, one sp2 quaternary carbon at *δ*_C_ 130.4, and one ester carbonyl at *δ*_C_ 166.0). In the ^1^H-NMR spectrum (Table 3), three methyl singlets (*δ*_H_ 1.02 s, 1.12 s, and 1.19 brs), one oxygenated methylene [*δ*_H_ 3.46 s (2H)] and one oxygenated methine [*δ*_H_ 3.46 s (2H) and 5.18 ddd, *J* = 4.2, 7.8, and 12.0 Hz] together with the multiple peaks of mono-substituted benzene protons (*δ*_H_ 7.45–7.98) distributed spread from upfield to downfield. Combining the information, compound **7** was reasoned to have a double-ring moiety in addition to a benzene and an ester carbonyl portion. A comprehensive analysis of ^1^H–^1^H COSY and HMBC spectra, an eudesmane-type sesquiterpene (Evans et al., 1982) was put forward by incorporating two COSY fragments (H_2_-1/H-2/H_2_-3 and H-5/H_2_-6/H-7/H_2_-8/H_2_-9) and the correlations of HMBC at CH_3_-14/C-1, C-5, C-9, and C-10 and CH_3_-15/C-3, C-4, and C-5. Moreover, the attachments of an *O*-benzoyl (OBz) group and a propane-1,2-diol group were confirmed to be at C-2 and C-7 by the HMBC correlations: H-2, H-2′, and H-3′ to ester carbonyl C-1′; H_2_-12 and H_3_-13 to C-7 and C-11 (Figure 4A). Concerning the stereochemistry of **7**, it was concluded to be a regular eudesmane-type sesquiterpene with anti-relationships of α-oriented H-5 and β-oriented CH_3_-14. Furthermore, both the cross peaks of NOESY correlations for H-2/H_β_-3, H-2/CH_3_-14, and H-2/CH_3_-15 suggested the β-orientation of H-2 and CH_3_-14 but α-orientation of H-7 given H_α_-3/H-5, and H-5/H-7 (Figure 4B). However, the conformation of C-11 was not unambiguous because it cannot be differentiated directly by the NOESY correlations of H-7/CH_3_-13, H_α_-8/CH_3_-13, H_β_-8/CH_3_-14, and H_β_-8/H_2_-12. For this reason, the dimolybdenum tetra-acetate [Mo_2_(OAc)_4_]-modified CD analysis was applied to resolve the steric assignment of the acyclic propane-1,2-diol group, a prim, sec-glycol (Frelek et al., 1997). As in many *vic*-glycols with a rigid conformation, one can follow the “helicity rule” to interpret the CD curves, whereby a positive (negative) torsional angle in the (HO)‒C‒C‒(OH) moiety should lead to a positive (negative) Cotton effect of 300 nm. In Figure 4C, a negative CD band at 300 nm is obvious and accompanied by a second Cotton effect of the same sign at around 400 nm corresponding to a negative torsional angle in the (HO)‒C‒C‒(OH) moiety. A Newman projection for the propane-1,2-diol moiety in chelation with Mo_2_(OAc)_4_ demonstrated a counterclockwise (a negative torsional angle) relationship from OH-12 to OH-11. Finally, compound **7** was clearly identified as 2-*O*-benzoyl-proximadiol, named mesoeudesmol B.

### 3.2 Effects of compounds 1‒7 on RAW264.7 macrophage cell viability and NO production

*M. procumbens* extracts have been shown with pharmacological potentialities for many inflammation-associated disorders (Huang et al., 2012; Huang et al., 2021). Given the isolated compounds **1**‒**7** from *M. procumbens*, we examined these isolates to see whether they similarly exhibit anti-inflammatory activities by using the LPS-induced RAW 264.7 macrophage cell model. As shown in Table 4, mesonaic acids D (**1**) and E (**2**), 2α,3α,19α-trihydroxy-24-norursa-4 (23),12-dien-28-oic acid (**6**), and mesoeudesmol B (**7**) show lower EC_50_ values than the positive control, quercetin (Li et al., 2016), suggesting that these four compounds possess better anti-inflammatory activity than those previously reported. Among them, mesoeudesmol B (**7**), a proximadiol derivative, displayed the highest inhibition activity on NO production with an EC_50_ value of 12.88 ± 0.23 μM than that of quercetin (an EC_50_ value of 24.12 ± 0.21 μM). Furthermore, all these seven compounds (at a concentration of 30 μΜ) showed an approximate 100% survival rate in cell viability assay, suggesting that they are, in general, safe with no major cytotoxicity (to RAW264.7 cells).

**TABLE 4:** Anti-NO production activity of compounds **1**‒**7**.

Compound	EC_50_ (μM)*^a^*	Cell viability (%)*^b^*
**1**	20.34 ± 0.61	102.57 ± 0.29
**2**	21.21 ± 0.52	101.43 ± 0.14
**3**	>30	103.31 ± 0.37
**4**	>30	99.41 ± 0.50
**5**	>30	100.94 ± 0.42
**6**	20.23 ± 0.12	101.52 ± 0.16
**7**	12.88 ± 0.23	102.56 ± 0.32
Quercetin*^c^*	24.12 ± 0.21	100.41 ± 0.53

a Cells were treated with LPS (1 μg) in combination with the test compound for 24 h.

b Cell viability was measured in the presence of 30 μM compound using the CCK-8 assay.

c Quercetin was used as a positive control.

### 3.3 Effects of compound 7 on the protein expression of iNOS and COX-2

**FIGURE 5:**
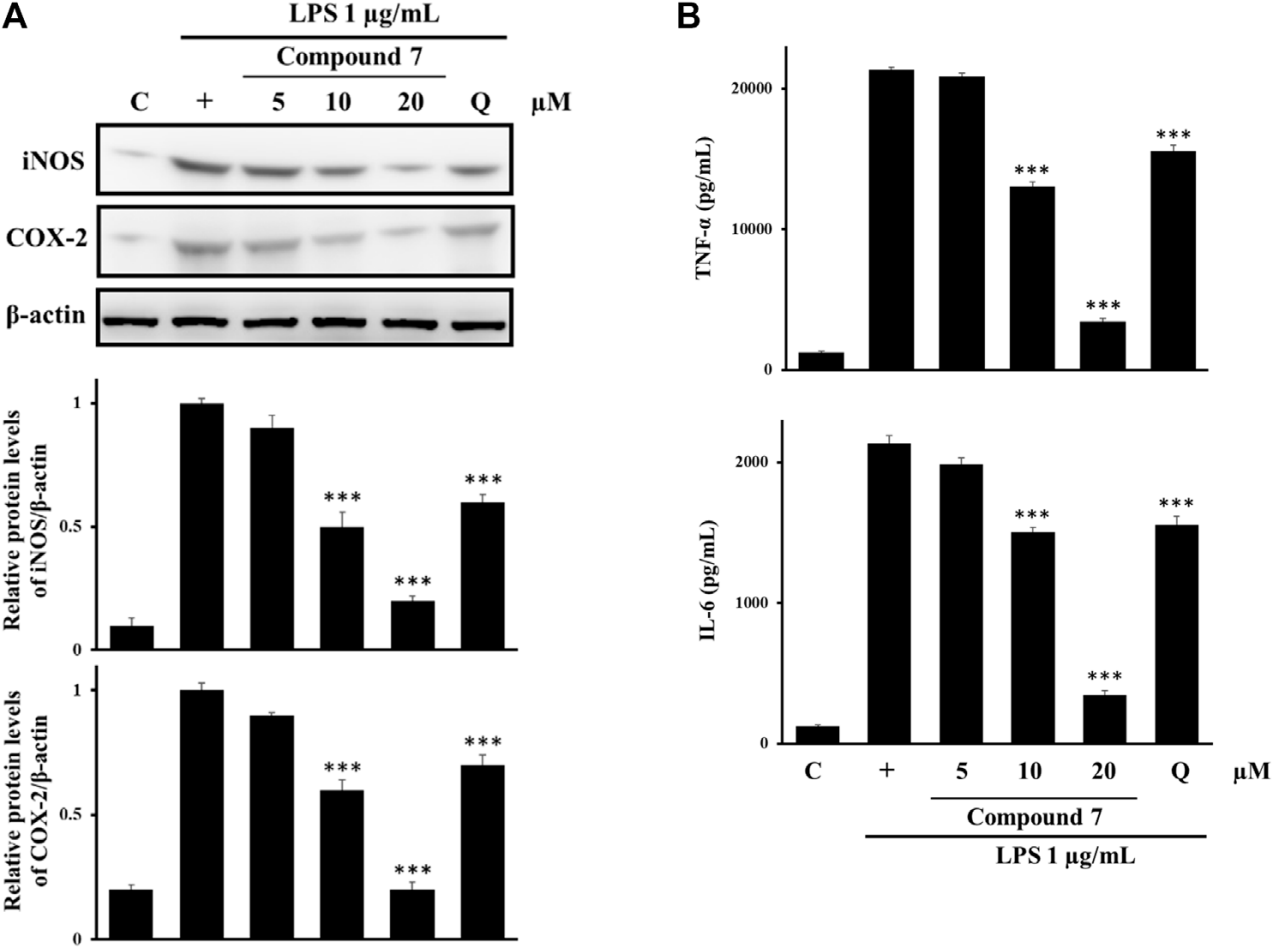
Effects of **7** on the expression of the iNOS and COX-2 proteins and cytokine secretion. RAW 264.7 cells were pretreated with the compounds for 1 h followed by stimulation with LPS (1 μg/ml) for an additional 24 h. **(A)** The expression of iNOS and COX-2 was determined using western blot analysis. The relative levels of iNOS and COX-2 were quantified by normalization to the β-actin levels. **(B)** The levels of TNF-α and IL-6 were measured using ELISA kits. Cells were treated with **7** at a concentration of 5–20 μM. Q: quercetin (25 μM). The data shown here represent the mean values of three independent experiments. ***p < 0.001 compared with the group treated with LPS.

To better understand the anti-inflammatory mechanism of compound **7**, we examined two key inflammation-mediated proteins in LPS-induced RAW264.7 cells, iNOS and COX-2 at the protein level (Murakami and Ohigashi, 2007). In terms of the expression level of iNOS and COX-2 shown in Figure 5A, the LPS-treated cells display a higher level as opposed to the non-treated cells, which show a low expression level. In addition, the protein level of the LPS-induced iNOS and COX-2 is negatively proportional to the addition of mesoeudesmol B (**7**) (5–20 μM) into the LPS-treated cells in a dose-dependent manner. Notably, 10 μM of **7** exhibits the lowest iNOS/COX-2 than 25 μM of quercetin does; moreover, the protein level of iNOS and COX-2 in the LPS-treated cells with the addition of **7** (20 μM) is nothing more than that in the non-treated cells, confirming that sesquiterpene **7** possesses stronger inhibitory activity than quercetin.

### 3.4 Effects of compound 7 on the secretion of cytokines TNF-α and IL-6

Given that TNF-α and IL-6 are major pro-inflammatory cytokines, they were further measured by ELISA in the LPS-stimulated murine macrophages. As shown in Figure 5B, it shows a dose-dependent suppression of the LPS-induced secretion of cytokines TNF-α and IL-6 with the addition of compound **7**, which shows a similar trend to that of the LPS-induced expression of iNOS and COX-2. Likewise, 10 μM of **7** exhibits a lower level of TNF-α and IL-6 than 25 μM of quercetin does; the level of TNF-α and IL-6 in the LPS-treated cells with the addition of **7** (20 μM) is close to the basal one, once again confirming that **7** is a stronger inflammatory inhibitor than quercetin.

## 4 Conclusion

Several lines of evidence have underscored that the phenolics (i.e. kaempferol and caffeic acid), triterpenoids (i.e. oleanolic acid and ursolic acid), and polysaccharides in *M. procumbens* Hemesley are functional ingredients with strong antioxidant and anti-inflammatory properties, thus making Hsian-tsao a superb heat-clearing (Qingre) and detoxifying (Jiedu) herb. Having gone through rigorous and comprehensive interrogations against the literature and chemical databases, the five triterpene acids (**1**–**5**) and one sesquiterpene (**7**) discovered herein are new chemical entities; mesoeudesmol B (**7**) featuring a proximadiol core structure, in particular, is the first of its kind isolated from the extract of *M. procumbens* Hemesley. Of them, three triterpene acids (**1**, **2,** and **6**) showed strong anti-inflammatory activities with the EC_50_ values of 20.3, 21.1, and 20.2 μM comparable to quercetin (EC_50_ values of 24.1 μM) and 15 triterpene acids isolated from our previous study, all presenting strong and promising anti-inflammatory potentials (Huang et al., 2021).

Of them, mesoeudesmol B (**7**), a 2-OBz proximadiol, outperformed others with an EC_50_ value of 12.9 μM 2-fold higher than quercetin in terms of anti-inflammation. Proximol^®^ (cryptomeridiol, a eudesmane sesquiterpenoid) is a well-known Egyptian folk medicine effective as a renal antispasmodic and diuretic agent extracted from the desert weed *Cymbopogon proximus* Stapf (Gramineae) but also from other species, such as *Dysphania graveolens* (Gui et al., 2020) and *Chenopodium vulvariap* (Locksley et al., 1982). This active sesquiterpene is best known for its high antidiabetic activity, whereas the *C. proximus* extracts are more versatile exhibiting multiple bioactivities, including antihypertensive activity and relaxation of the smooth muscle fibers (El-Nezhawy et al., 2014).

We believe that mesoeudesmol B (**7**) is the dominant agent in *M. procumbens*. The anti-inflammation mechanism of **7** is that it suppresses inflammation-mediated proteins (iNOS and COX-2) and pro-inflammatory cytokines (TNF-α and IL-6), thereby underscoring Hsian-tsao (the grass jelly herb) a superb medicinal herb worth of further studies for advanced pharmacologic applications and biosynthetic diversifications.

## Data Availability

The datasets presented in this study can be found in online repositories. The names of the repository/repositories and accession number(s) can be found in the article/Supplementary Material.
