# Supplementary material for: Anti-inflammatory sesquiterpene and triterpene acids from Mesona procumbens Hemsley
Source: Front Chem. 2022 Sep 15;10:1003356. doi: 10.3389/fchem.2022.1003356 (PMC9520569; doi:10.3389/fchem.2022.1003356)
Supplement: Supplementary file 1 [file DataSheet1.PDF]

Supplementary Material

Figure S1.1. ^{1}H-NMR spectrum of compound **1** in methanol-d_4 (600 MHz).

Figure S1.2. ^{13}C-NMR spectrum of compound **1** in methanol-d_4 (150 MHz).

Figure S1.3. HSQC spectrum of compound **1**.

Figure S1.4. HSQC spectrum of compound **1**.

Figure S1.5. HMBC spectrum of compound **1**.

Figure S1.6. NOESY spectrum of compound **1**.

Figure S1.7. HRESIMS of compound **1**.

Figure S2.1. ^{1}H-NMR spectrum of compound **2** in methanol-d_4 (600 MHz).

Figure S2.2. ^{13}C-NMR spectrum of compound **2** in methanol-d_4 (150 MHz).

Figure S2.3. HSQC spectrum of compound **2**.

Figure S2.4. HSQC spectrum of compound **2**.

Figure S2.5. HMBC spectrum of compound **2**.

Figure S2.6. NOESY spectrum of compound **2**.

Figure S2.7. HRESIMS of compound **2**.

Figure S3.1. ^{1}H-NMR spectrum of compound **3** in methanol-d_4 (600 MHz).

Figure S3.2. ^{13}C-NMR spectrum of compound **3** in methanol-d_4 (150 MHz).

Figure S3.3. HSQC spectrum of compound **3**.

Figure S3.4. HSQC spectrum of compound **3**.

Figure S3.5. HMBC spectrum of compound **3**.

Figure S3.6. NOESY spectrum of compound **3**.

Figure S3.7. HRESIMS of compound **3**.

Figure S4.1. ^{1}H-NMR spectrum of compound **4** in methanol-d_4 (600 MHz).

Figure S4.2. ^{13}C-NMR spectrum of compound **4** in methanol-d_4 (150 MHz).

Figure S4.3. HSQC spectrum of compound **4**.

Figure S4.4. HSQC spectrum of compound **4**.

Figure S4.5. HMBC spectrum of compound **4**.

Figure S4.6. NOESY spectrum of compound **4**.

Figure S4.7. HRESIMS of compound **4**.

Figure S5.1. ^{1}H-NMR spectrum of compound **5** in methanol-d_4 (500 MHz).

Figure S5.2. ^{13}C-NMR spectrum of compound **5** in methanol-d_4 (125 MHz).

Figure S5.3. HSQC spectrum of compound **5**.

Figure S5.4. HSQC spectrum of compound **5**.

Figure S5.5. HMBC spectrum of compound **5**.

Figure S5.6. NOESY spectrum of compound **5**.

Figure S5.7. HRESIMS of compound **5**.

Figure S6.1. ^{1}H-NMR spectrum of compound **6** in methanol-d_4 (600 MHz).

Figure S6.2. ^{13}C-NMR spectrum of compound **6** in methanol-d_4 (150 MHz).

Figure S6.3. HSQC spectrum of compound **6**.

Figure S6.4. HSQC spectrum of compound **6**.

Figure S6.5. HMBC spectrum of compound **6**.

Figure S6.6. NOESY spectrum of compound **6**.

Figure S6.7. HRESIMS of compound **6**.

Figure S7.1. ^{1}H-NMR spectrum of compound **7** in methanol-d_4 (600 MHz).

Figure S7.2. ^{13}C-NMR spectrum of compound **7** in methanol-d_4 (150 MHz).

Figure S7.3. HSQC spectrum of compound **7**.

Figure S7.4. HSQC spectrum of compound **7**.

Figure S7.5. HMBC spectrum of compound **7**.

Figure S7.6. NOESY spectrum of compound **7**.

Figure S7.7. HRESIMS of compound **7**.

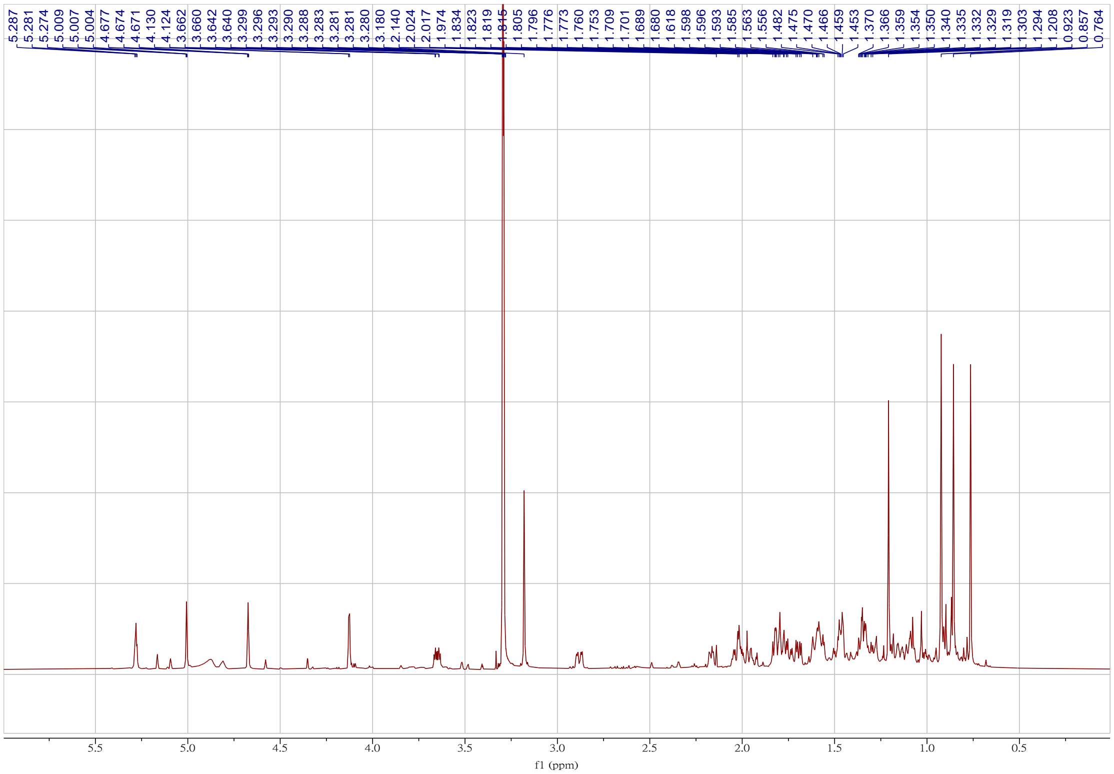

Figure S1.1. ^{1}H-NMR spectrum of compound **1** in methanol-d_4 (600 MHz).

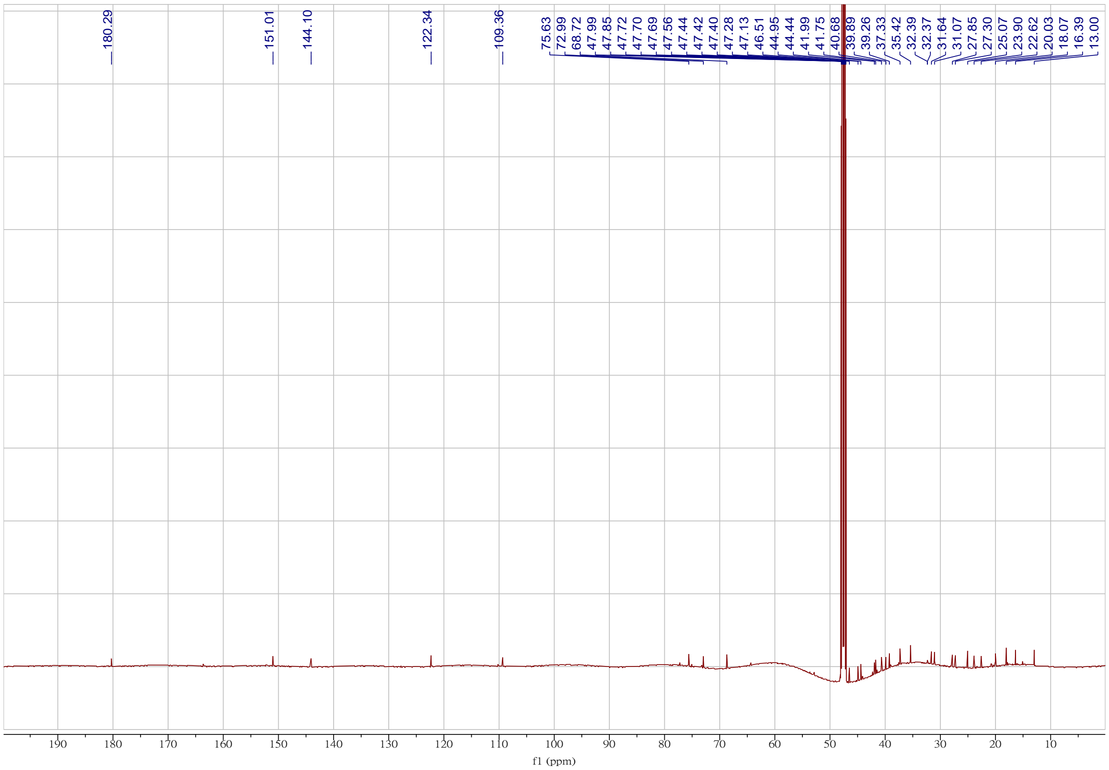

Figure S1.2. ^{13}C-NMR spectrum of compound **1** in methanol-d_4 (150 MHz).

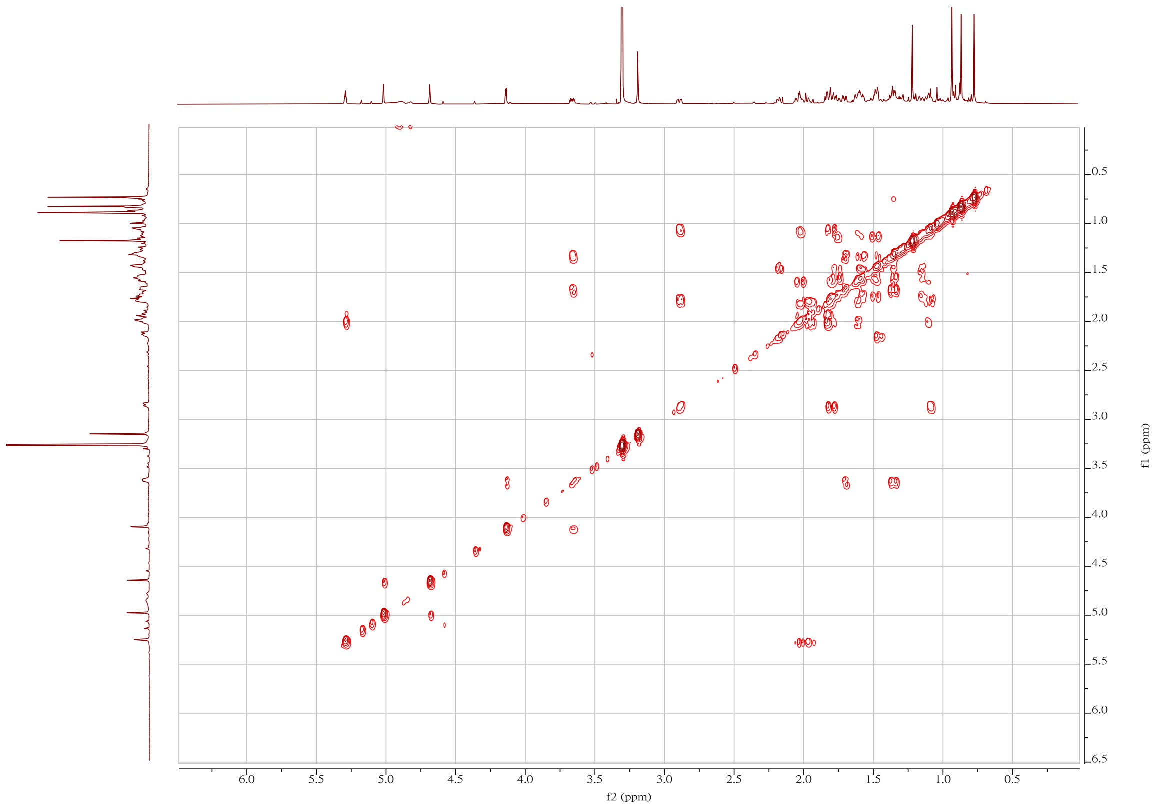

Figure S1.3. ^{1}H-^{1}H COSY spectrum of compound **1**.

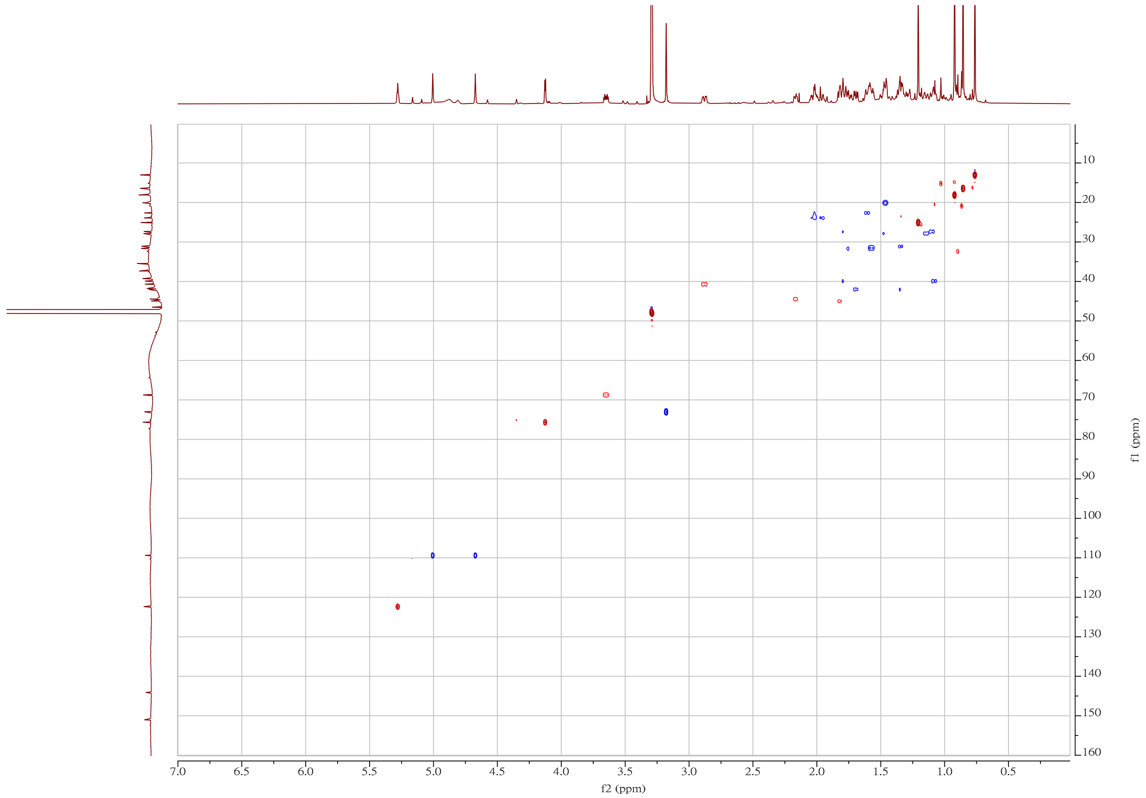

Figure S1.4. HSQC spectrum of compound **1**.

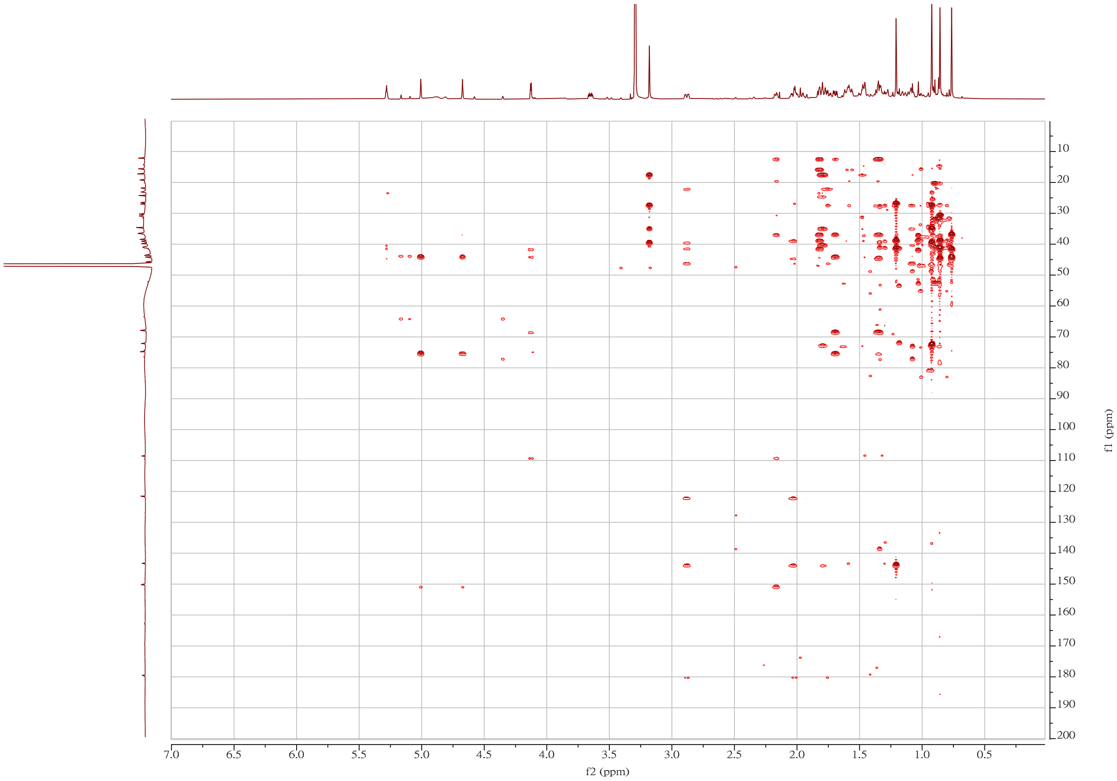

Figure S1.5. HMBC spectrum of compound **1**.

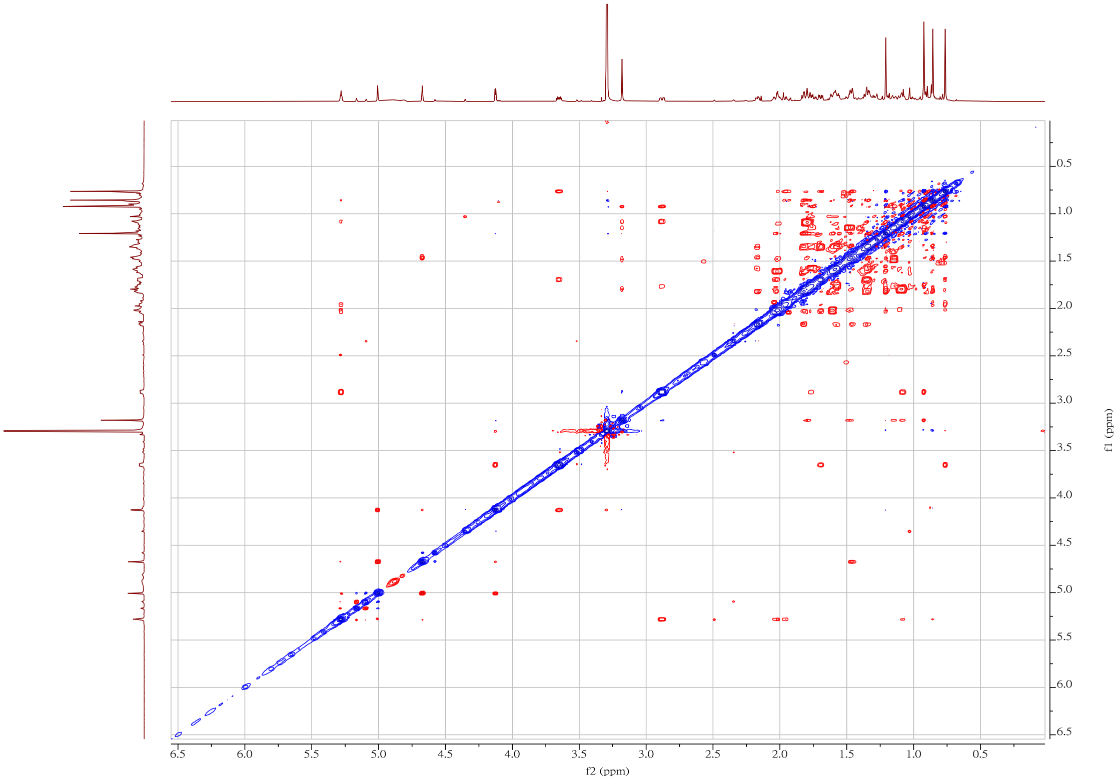

Figure S1.6. NOESY spectrum of compound **1**.

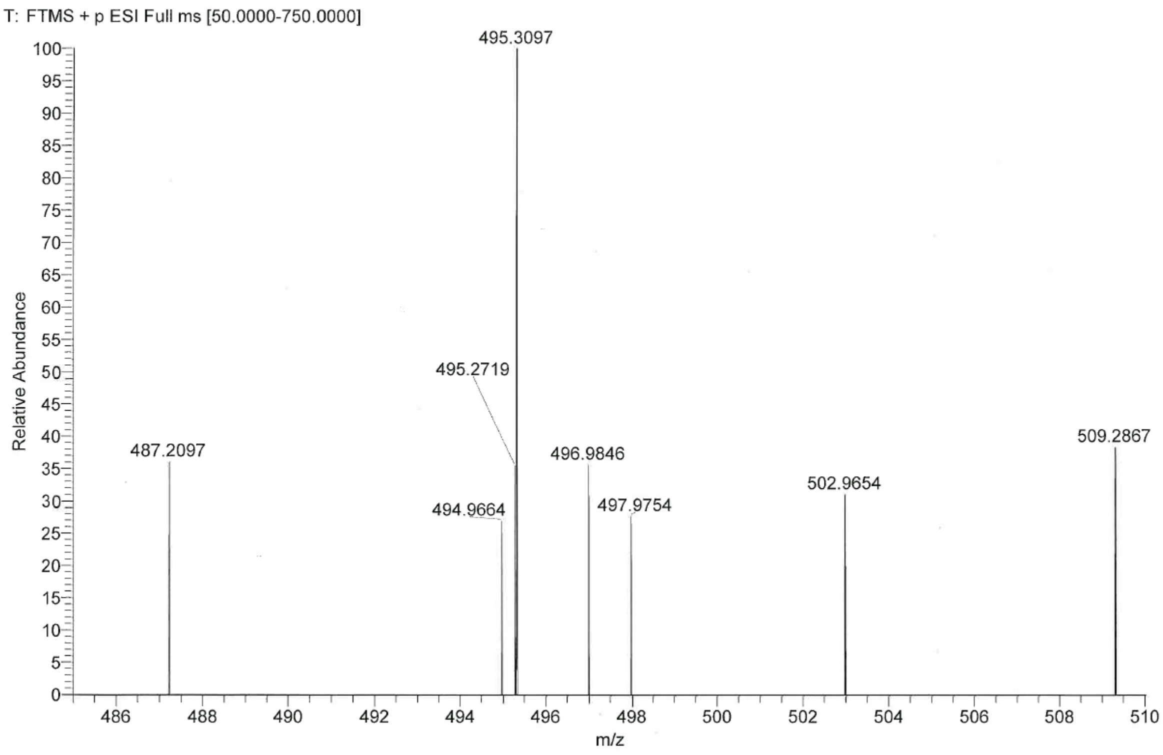

Figure S1.7. HRESIMS of compound **1**.

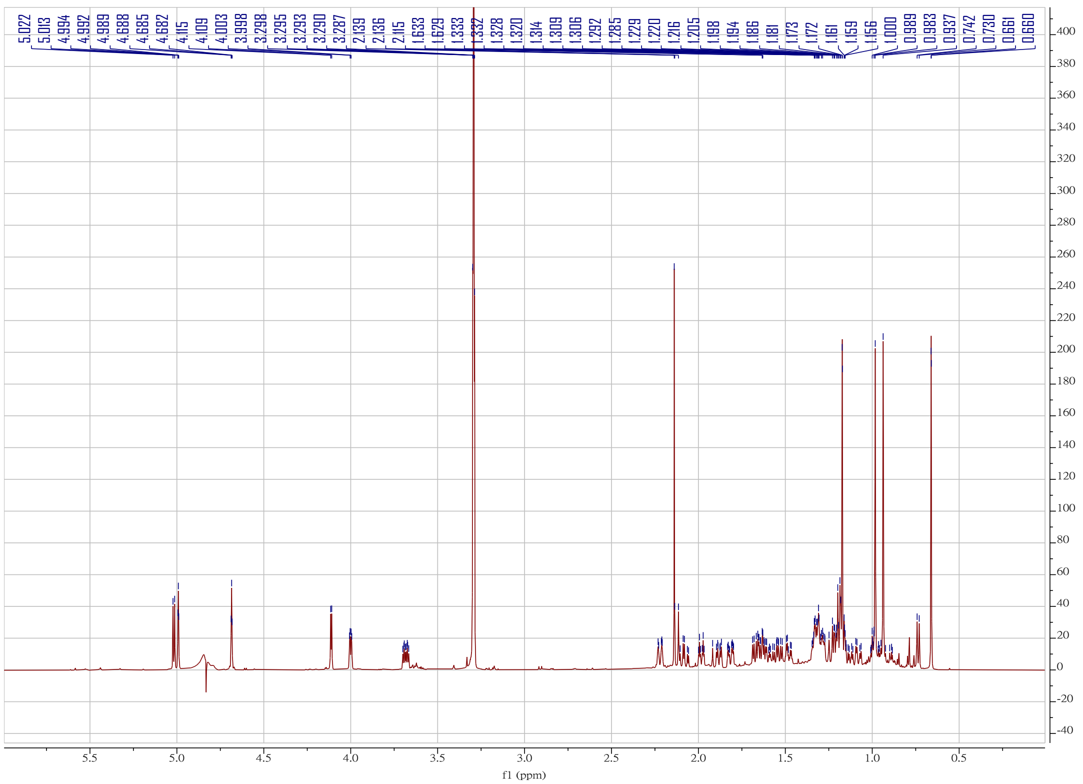

Figure S2.1. ^{1}H-NMR spectrum of compound **2** in methanol-d_4 (600 MHz).

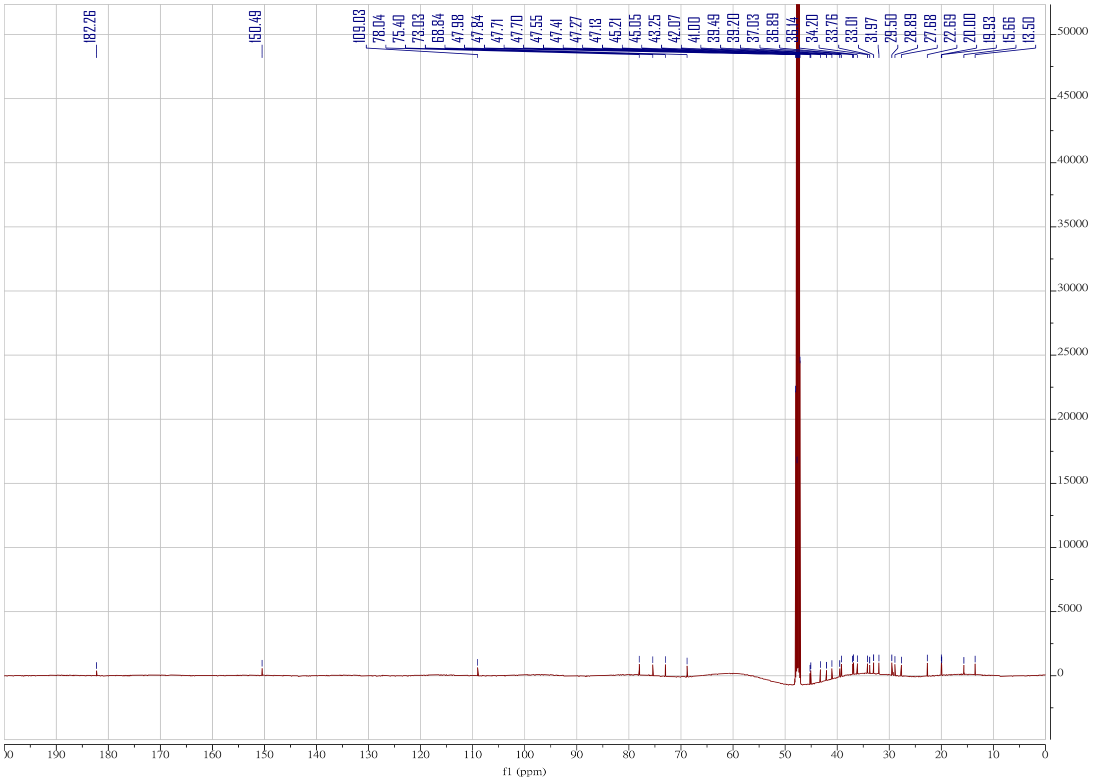

Figure S2.2. ^{13}C-NMR spectrum of compound **2** in methanol-d_4 (150 MHz).

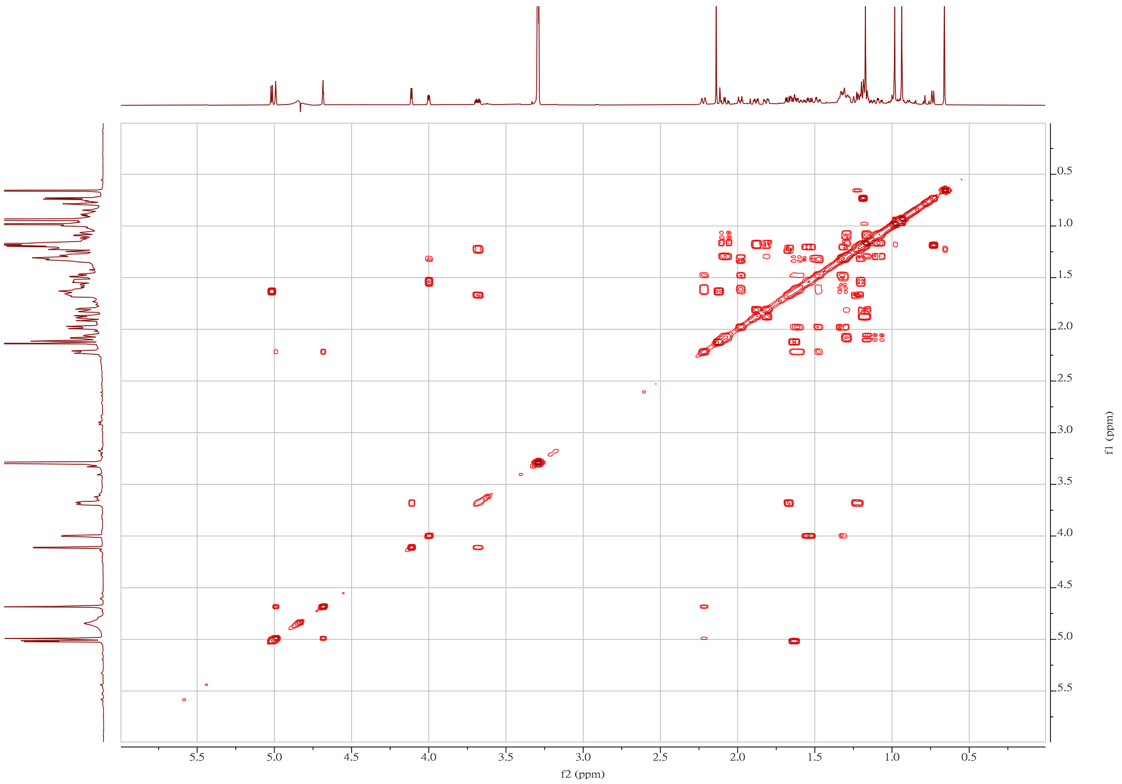

Figure S2.3. ^{1}H-^{1}H COSY spectrum of compound **2**.

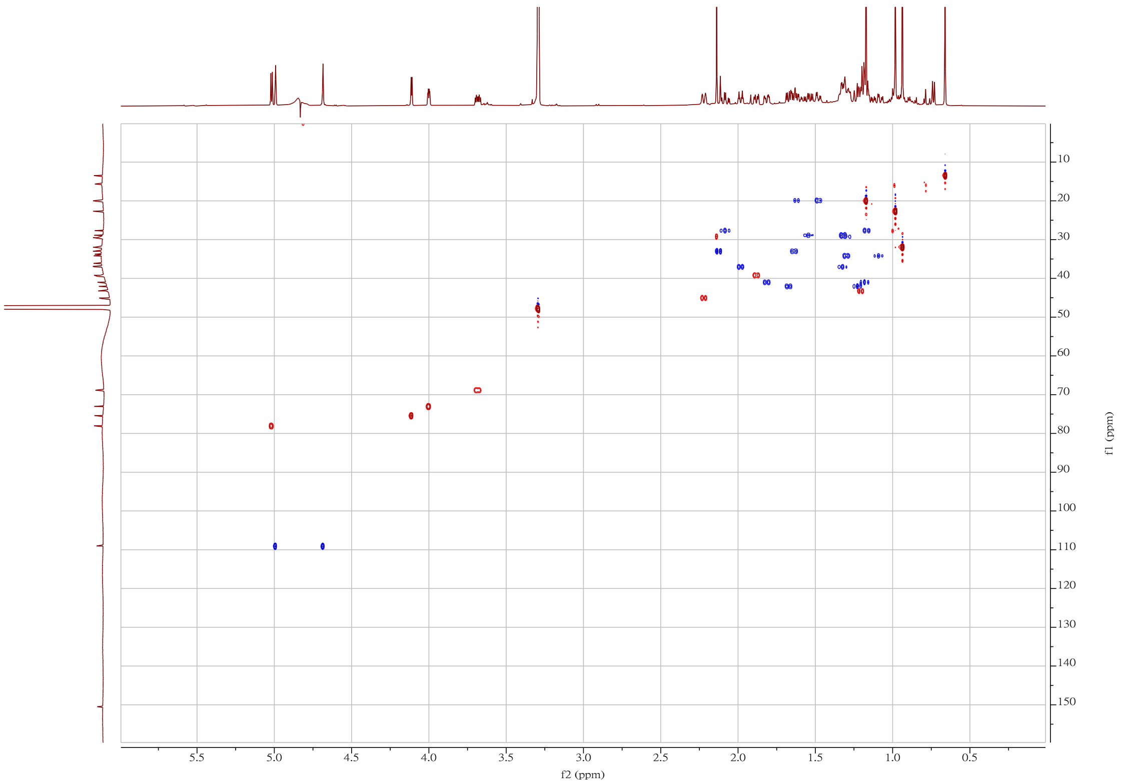

Figure S2.4. HSQC spectrum of compound **2**.

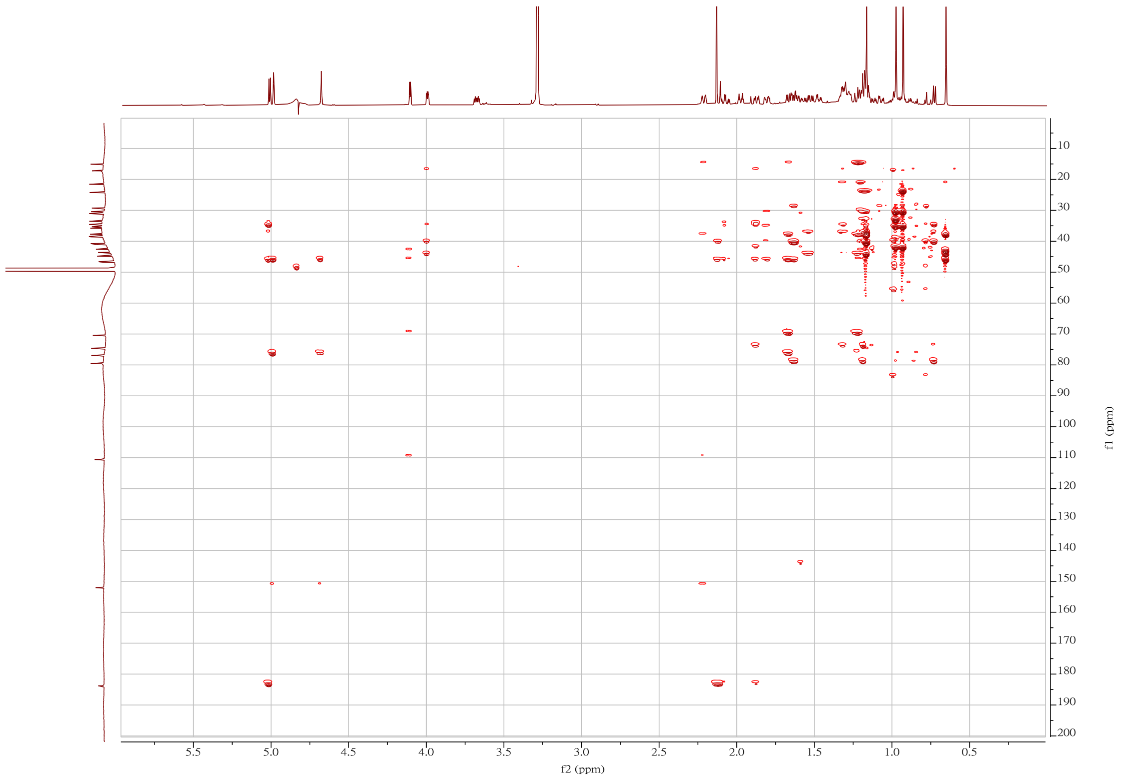

Figure S2.5. HMBC spectrum of compound **2**.

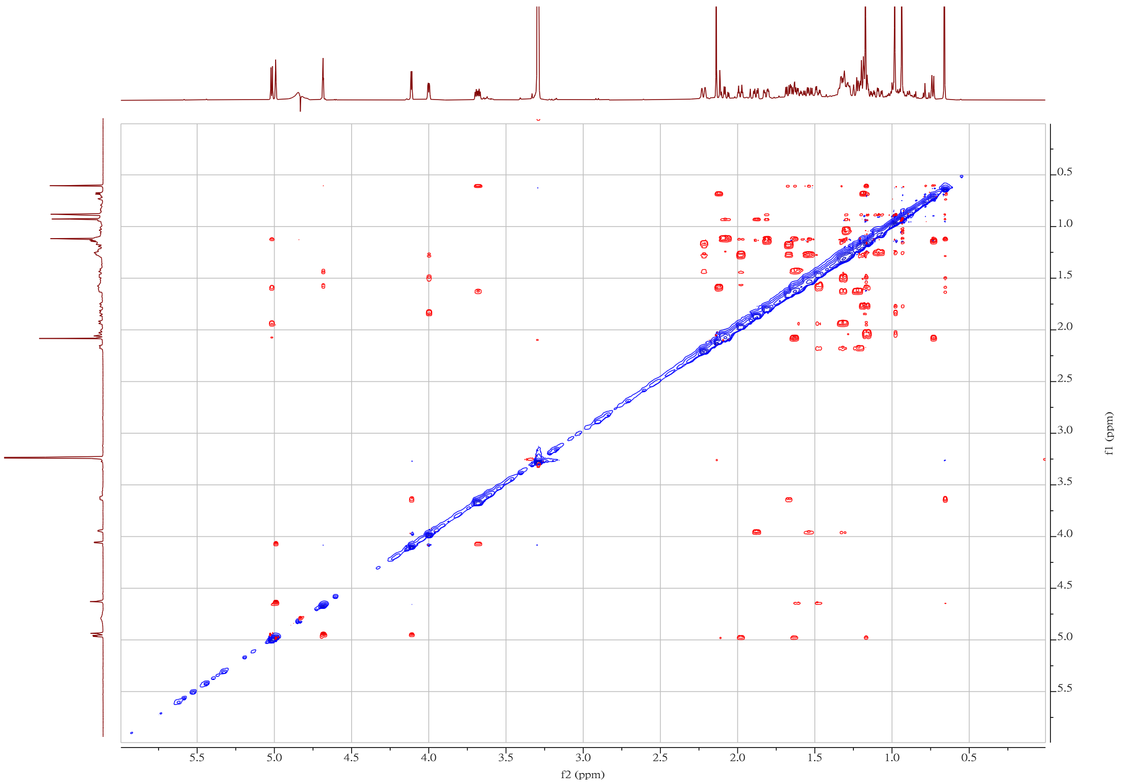

Figure S2.6. NOESY spectrum of compound **2**.

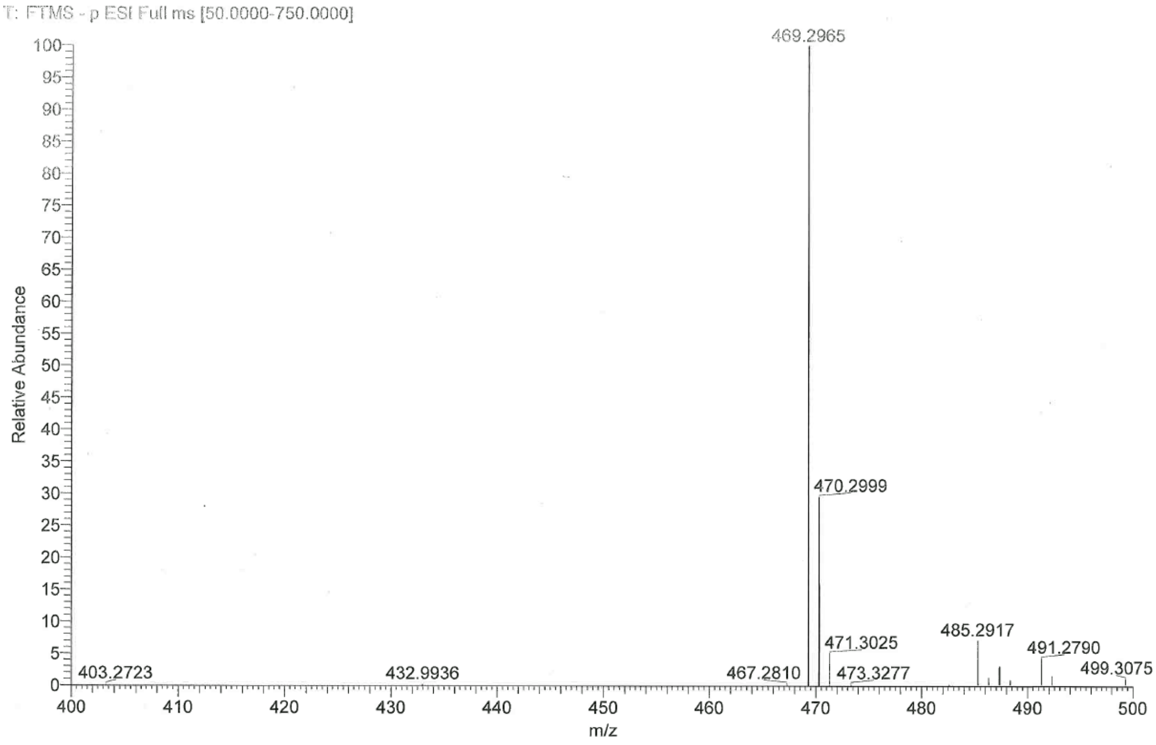

Figure S2.7. HRESIMS of compound **2**.

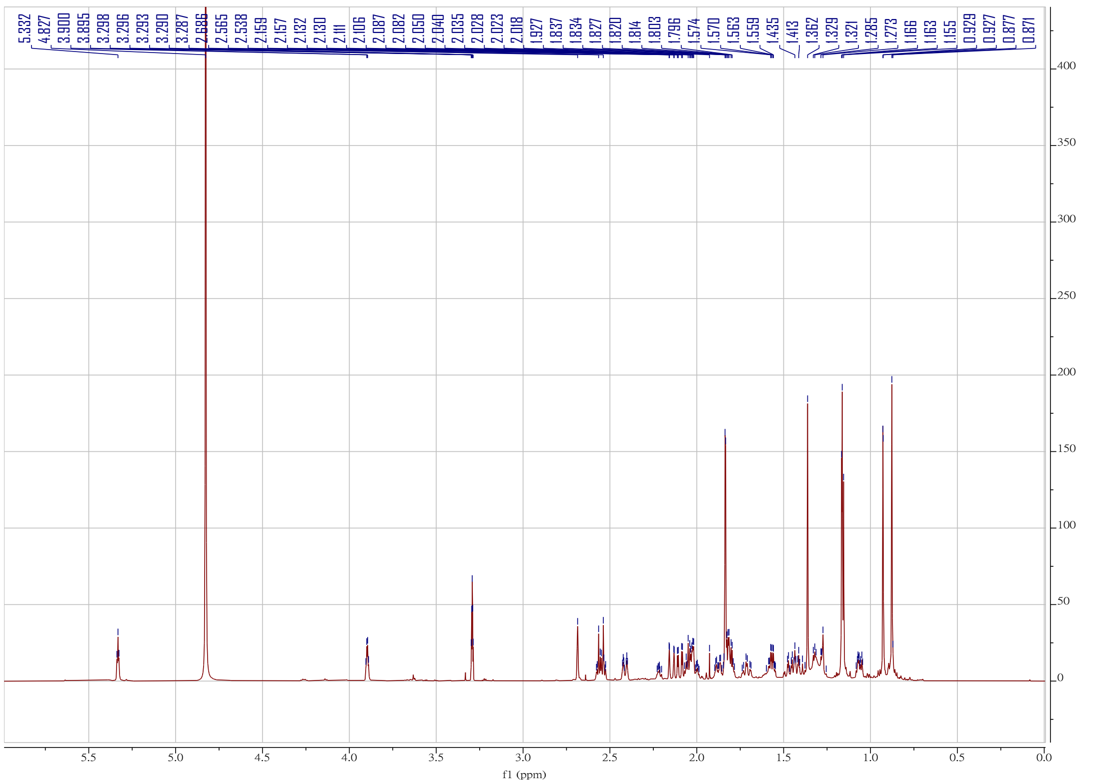

Figure S3.1. ^{1}H-NMR spectrum of compound **3** in methanol-d_4 (600 MHz).

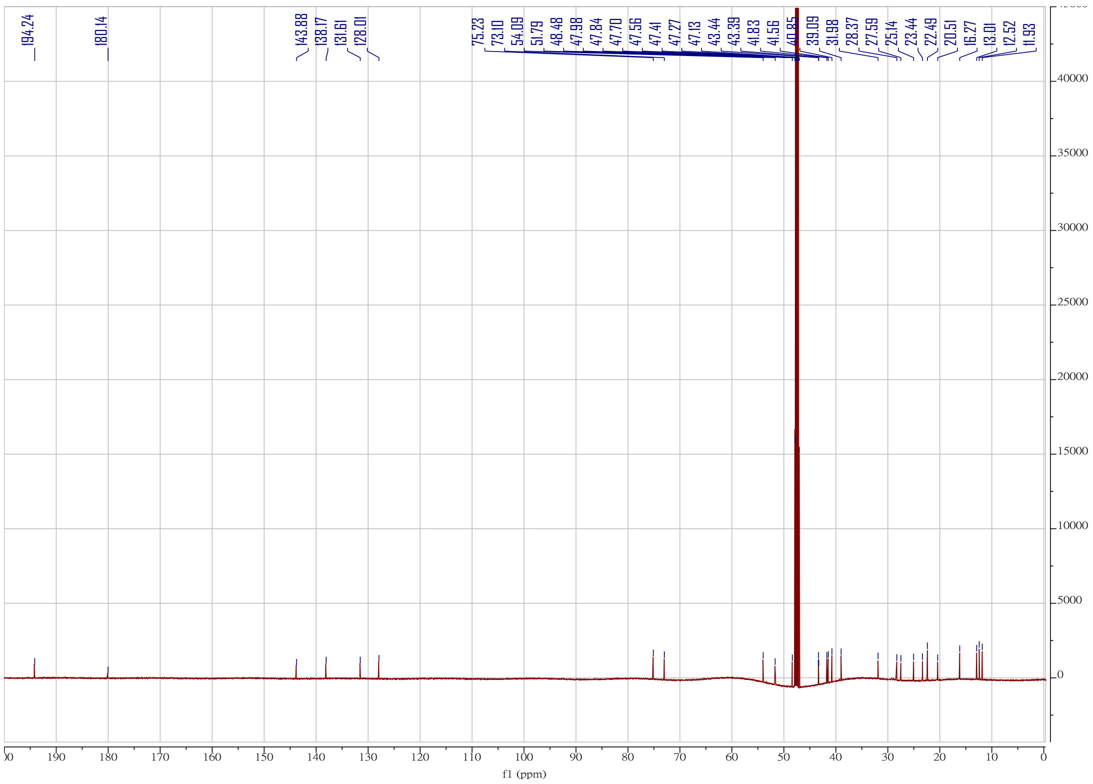

Figure S3.2. ^{13}C-NMR spectrum of compound **3** in methanol-d_4 (150 MHz).

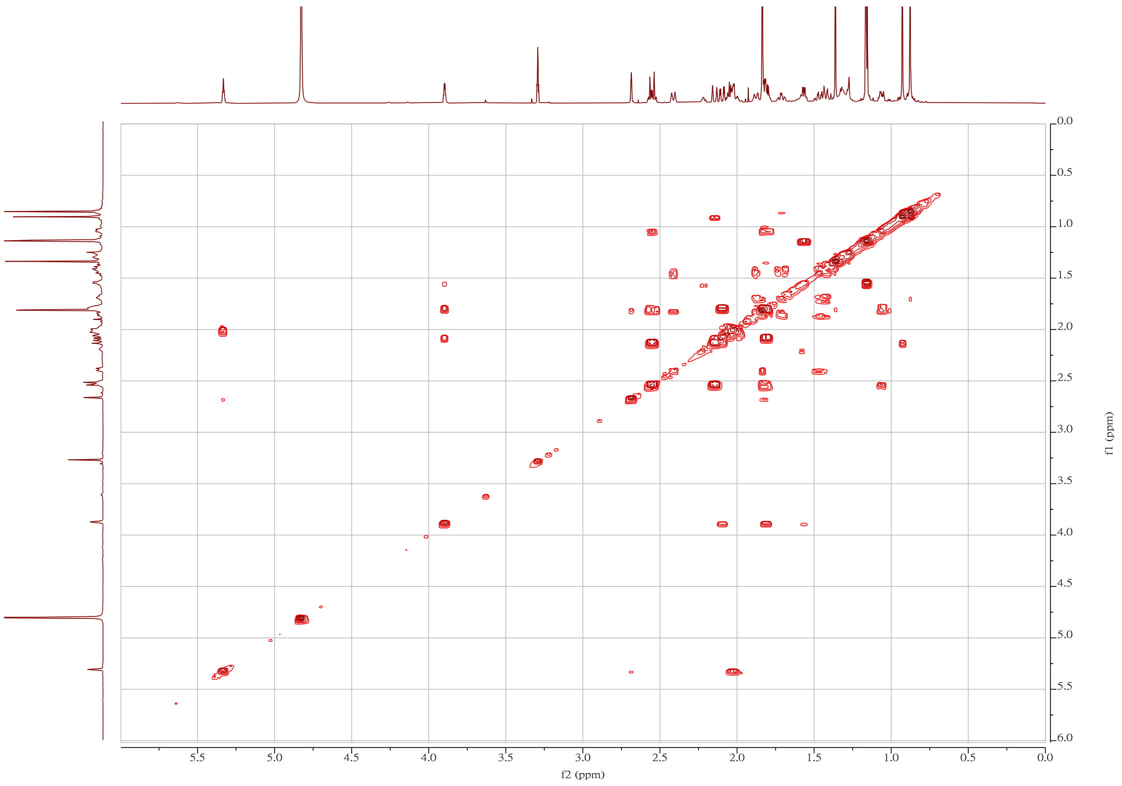

Figure S3.3. ^{1}H-^{1}H spectrum of compound **3**.

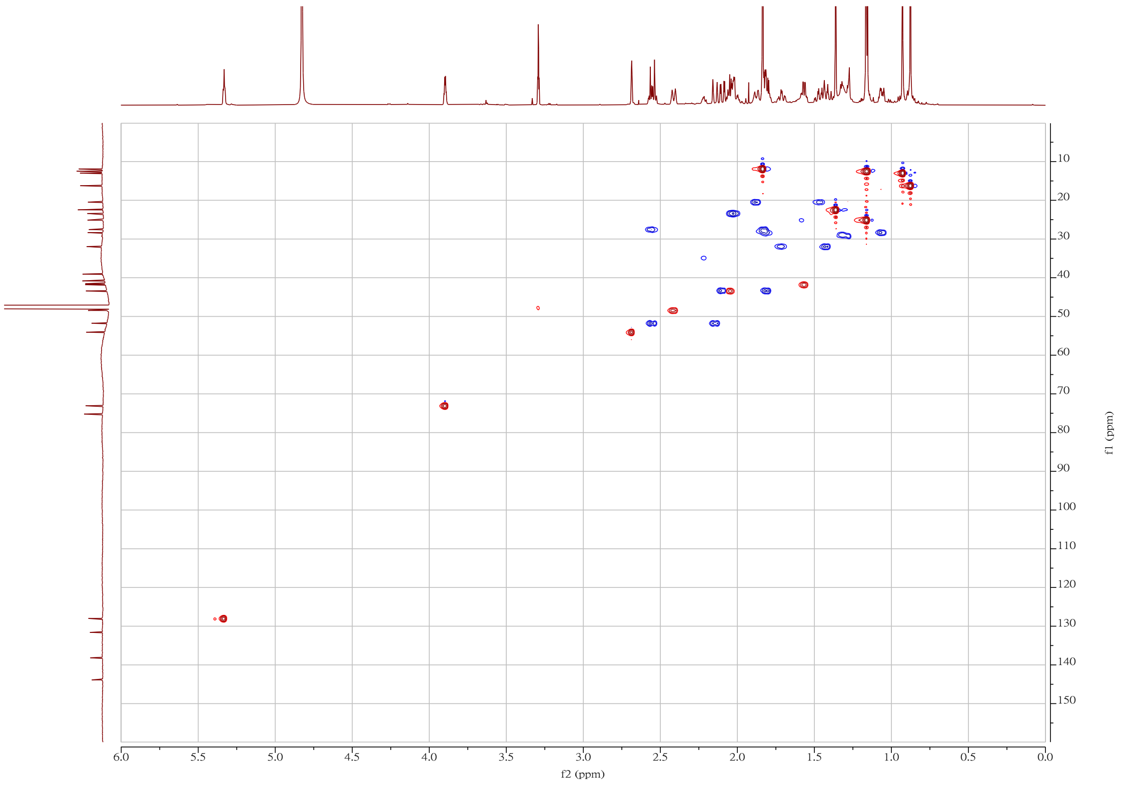

Figure S3.4. HSQC spectrum of compound **3**.

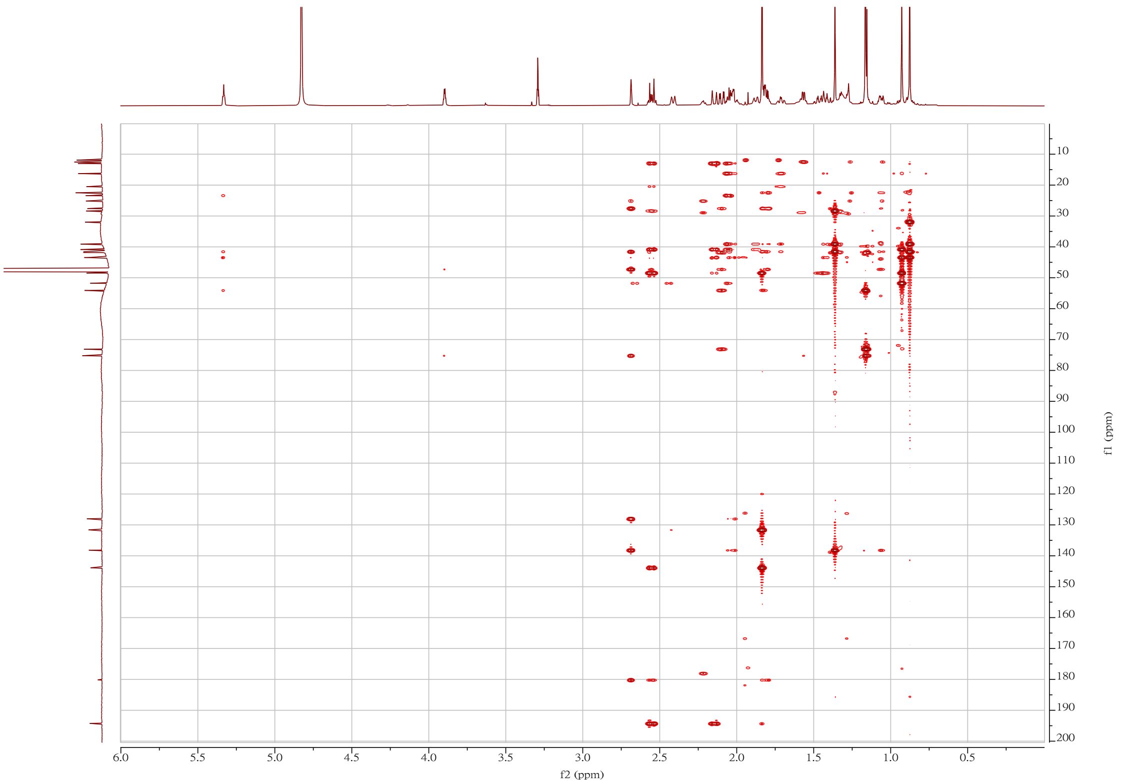

Figure S3.5. HMBC spectrum of compound **3**.

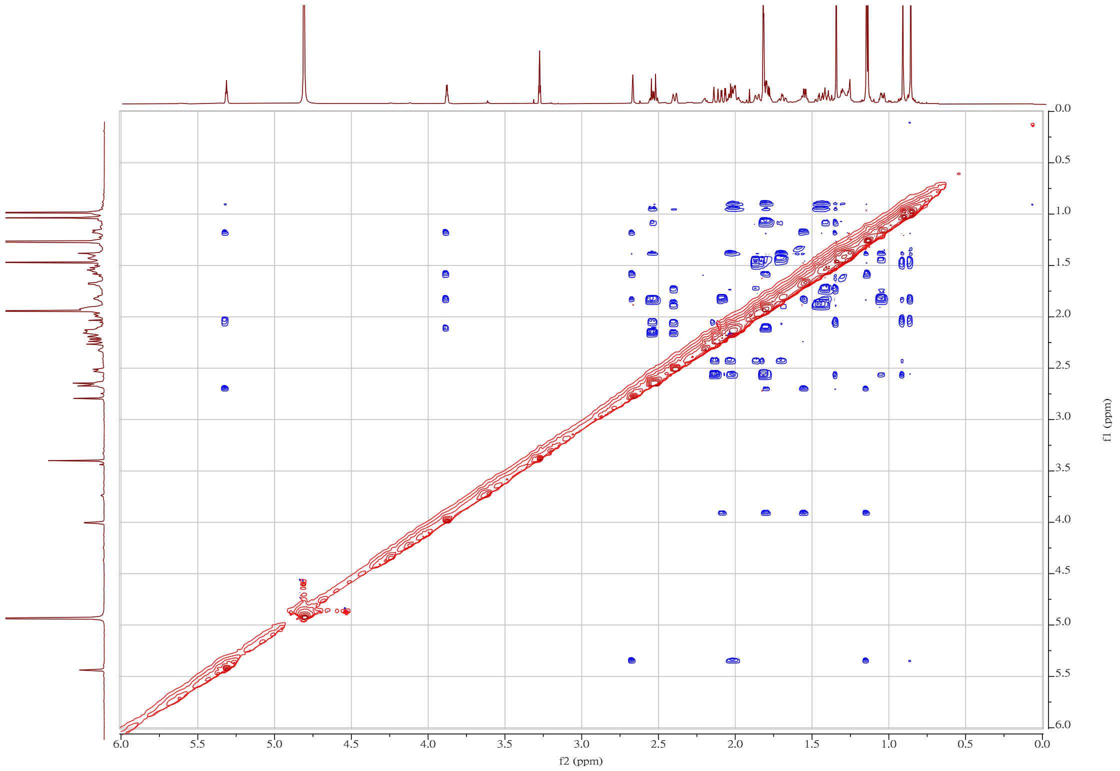

Figure S3.6. NOESY spectrum of compound **3**.

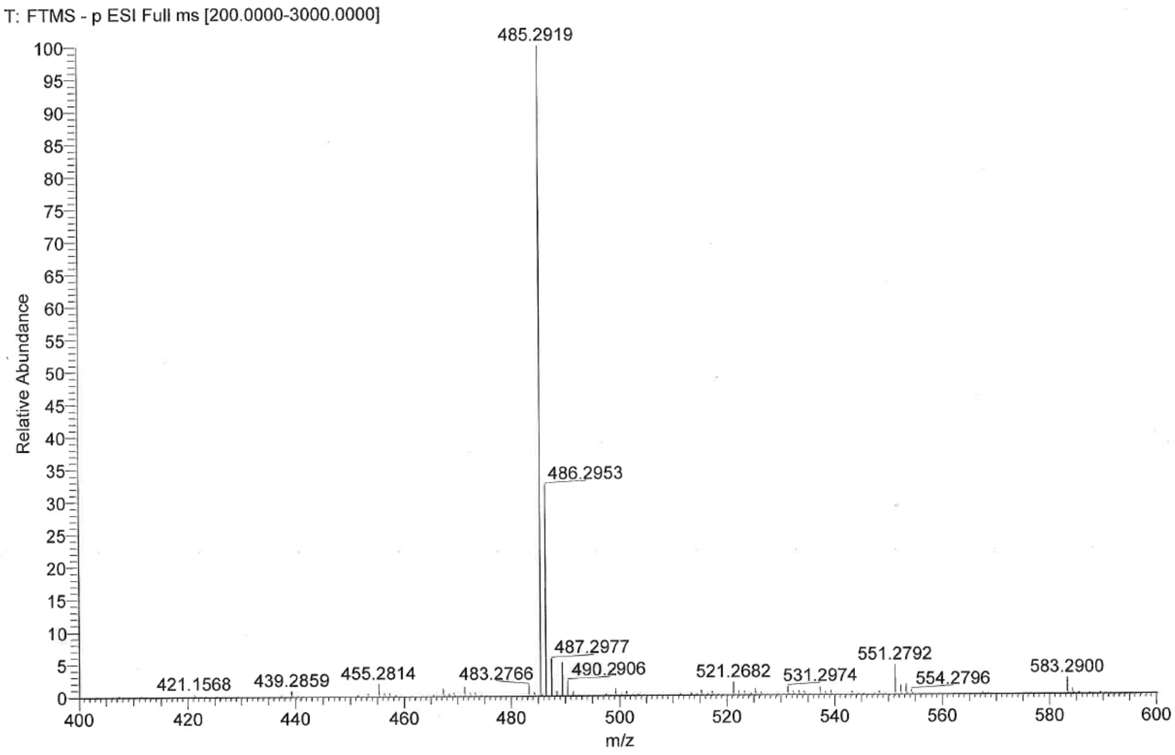

Figure S3.7. HRESIMS of compound **3**.

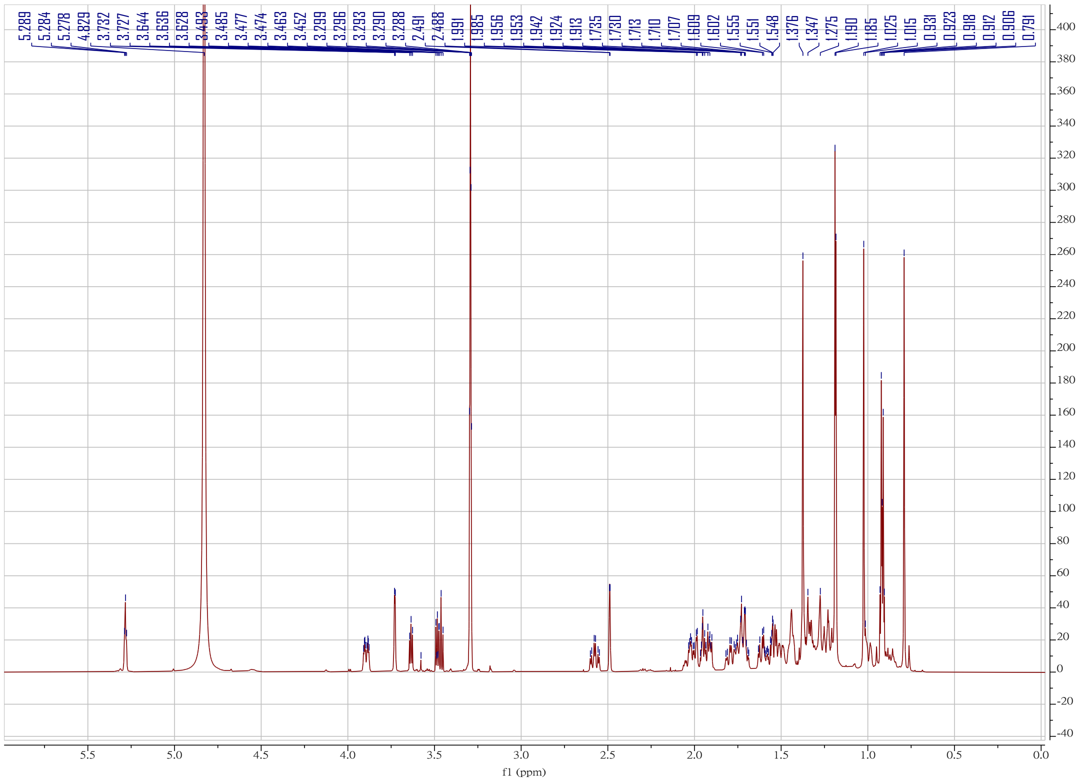

Figure S4.1. ^{1}H-NMR spectrum of compound **4** in methanol-d_4 (600 MHz).

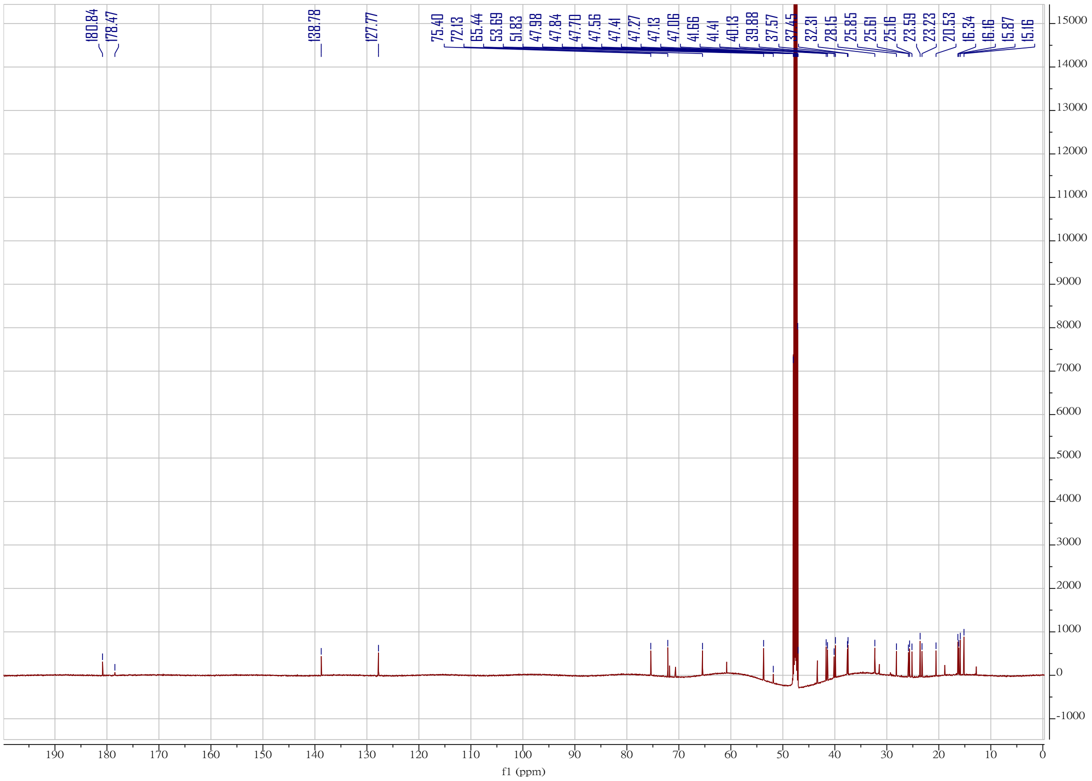

Figure S4.2. ^{13}C-NMR spectrum of compound **4** in methanol-d_4 (150 MHz).

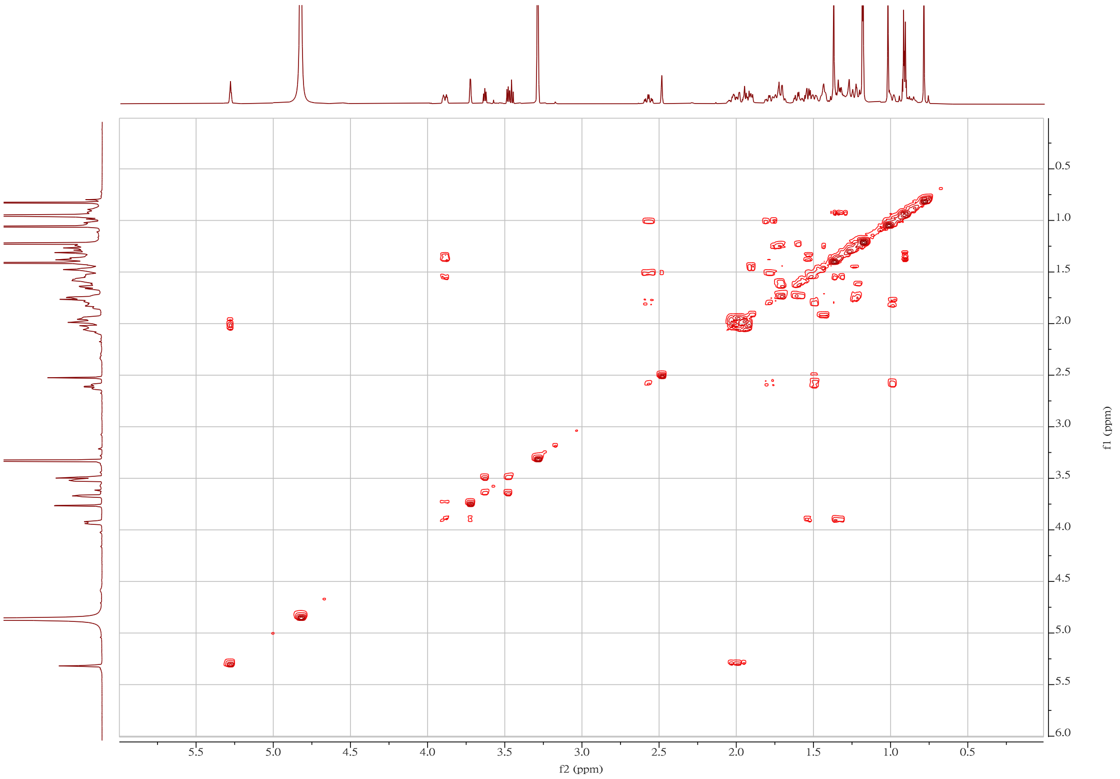

Figure S4.3. ^{1}H-^{1}H spectrum of compound **4**.

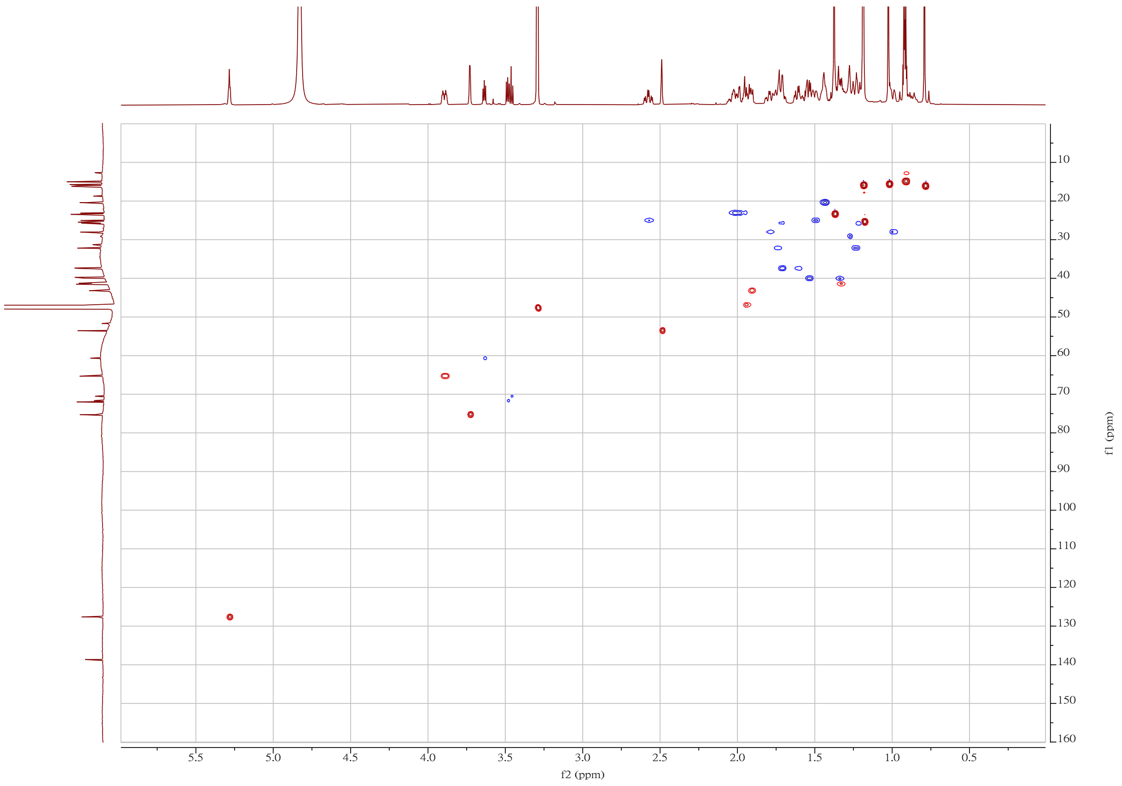

Figure S4.4. HSQC spectrum of compound **4**.

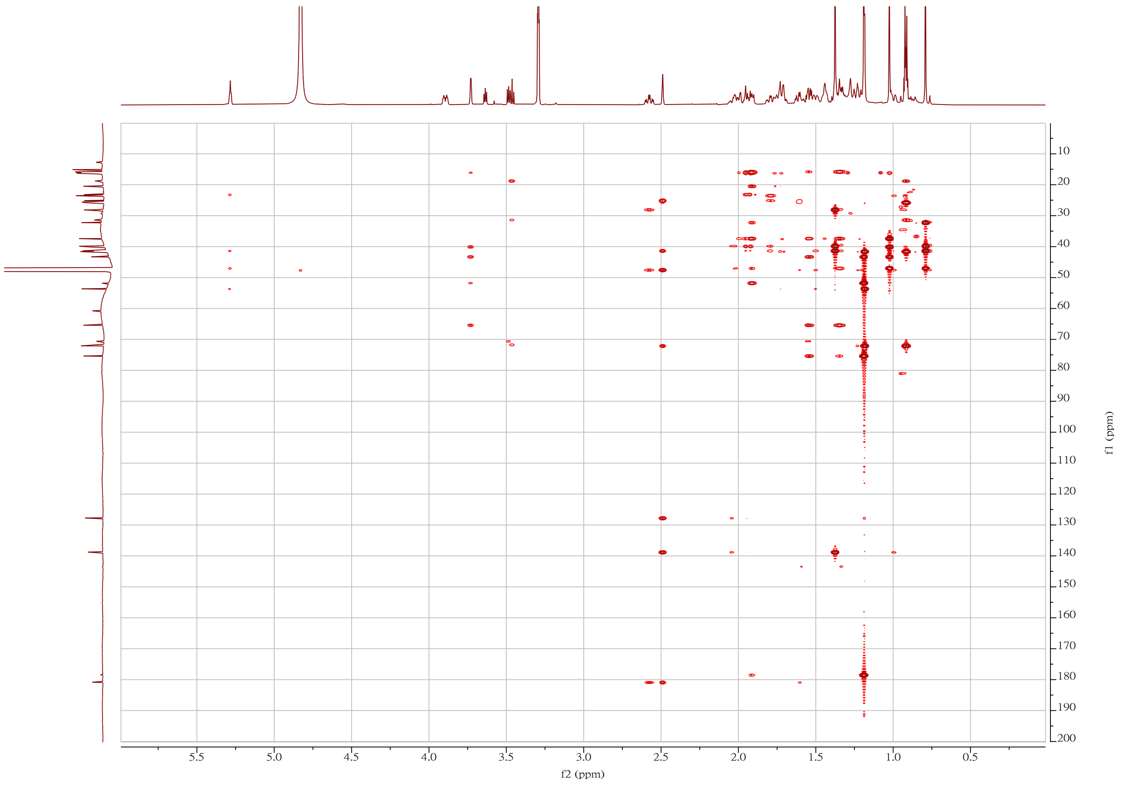

Figure S4.5. HMBC spectrum of compound **4**.

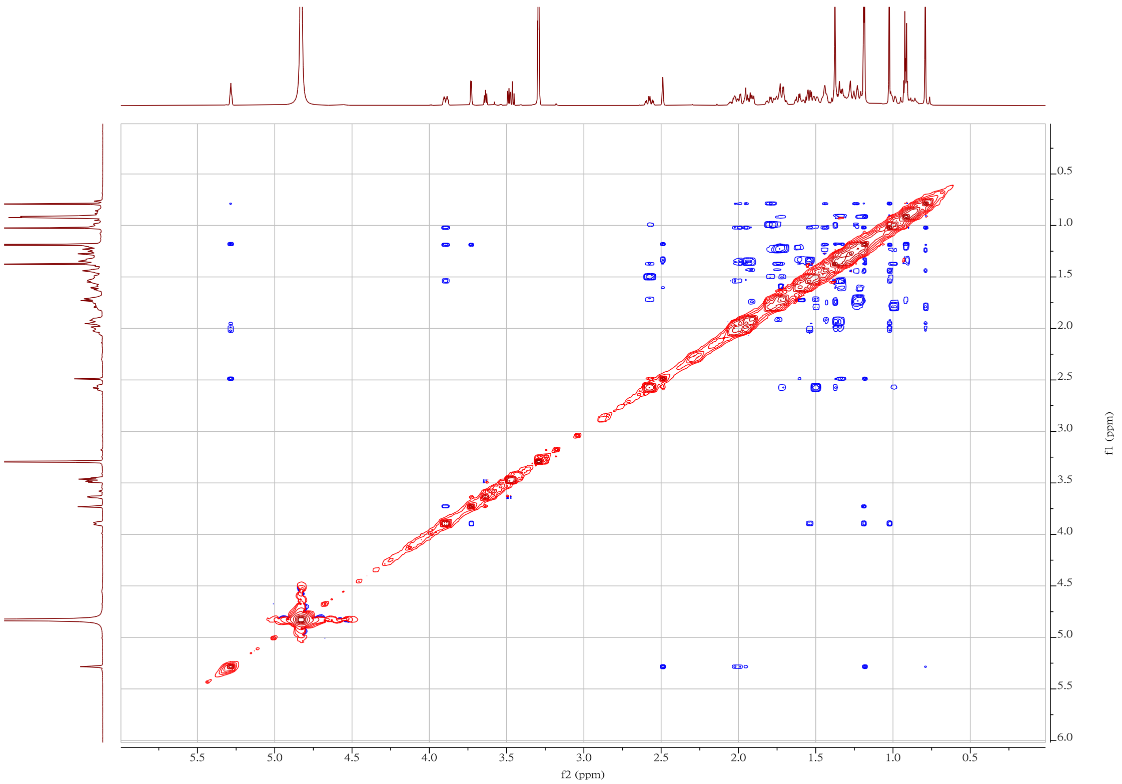

Figure S4.6. NOESY spectrum of compound **4**.

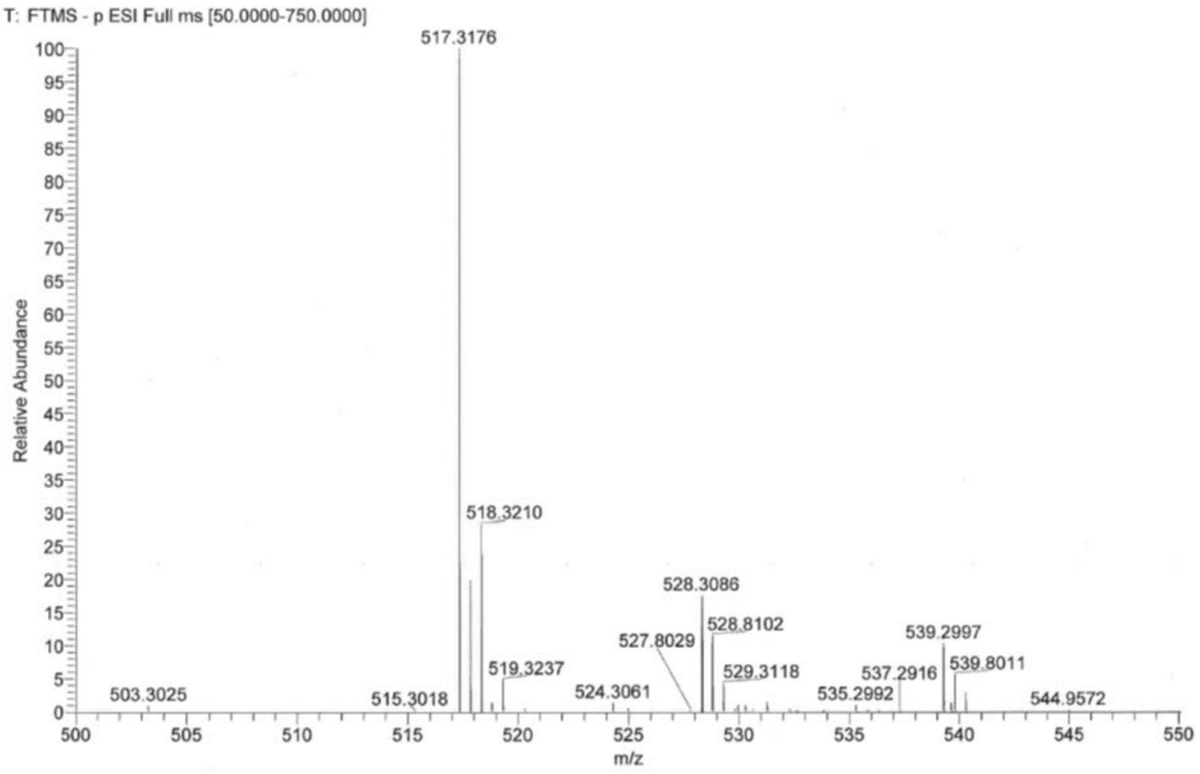

Figure S4.7. HRESIMS of compound **4**.

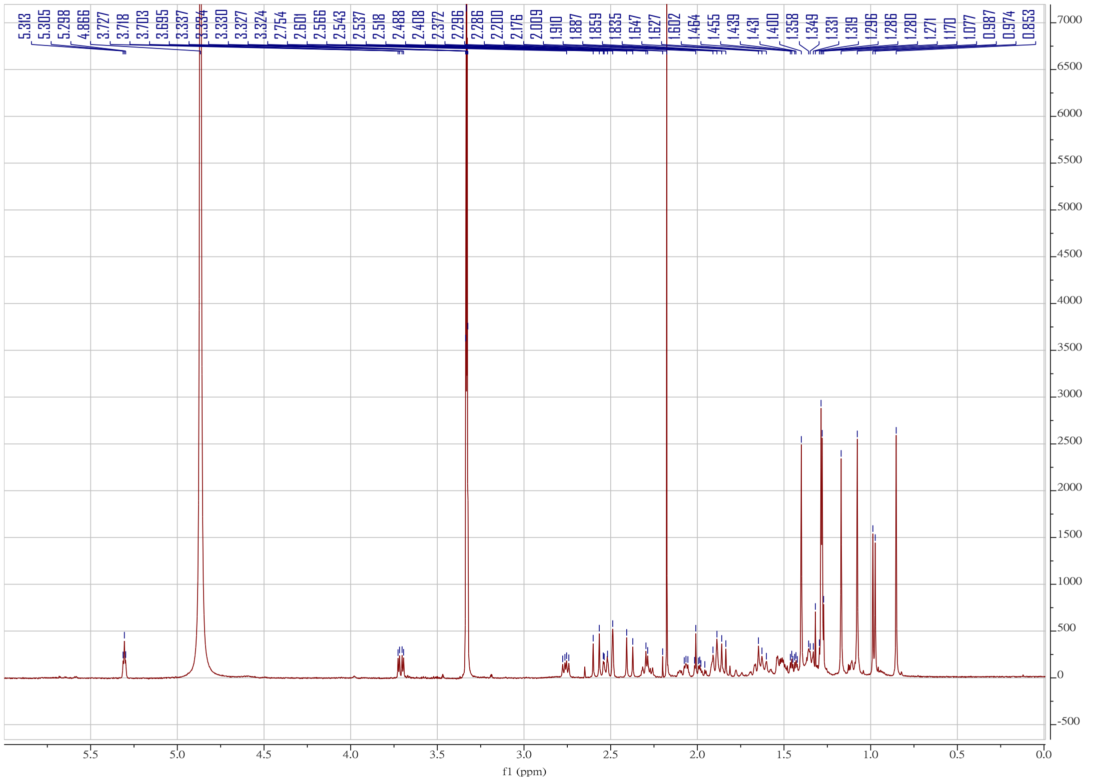

Figure S5.1. ^{1}H-NMR spectrum of compound **5** in methanol-d_4 (500 MHz).

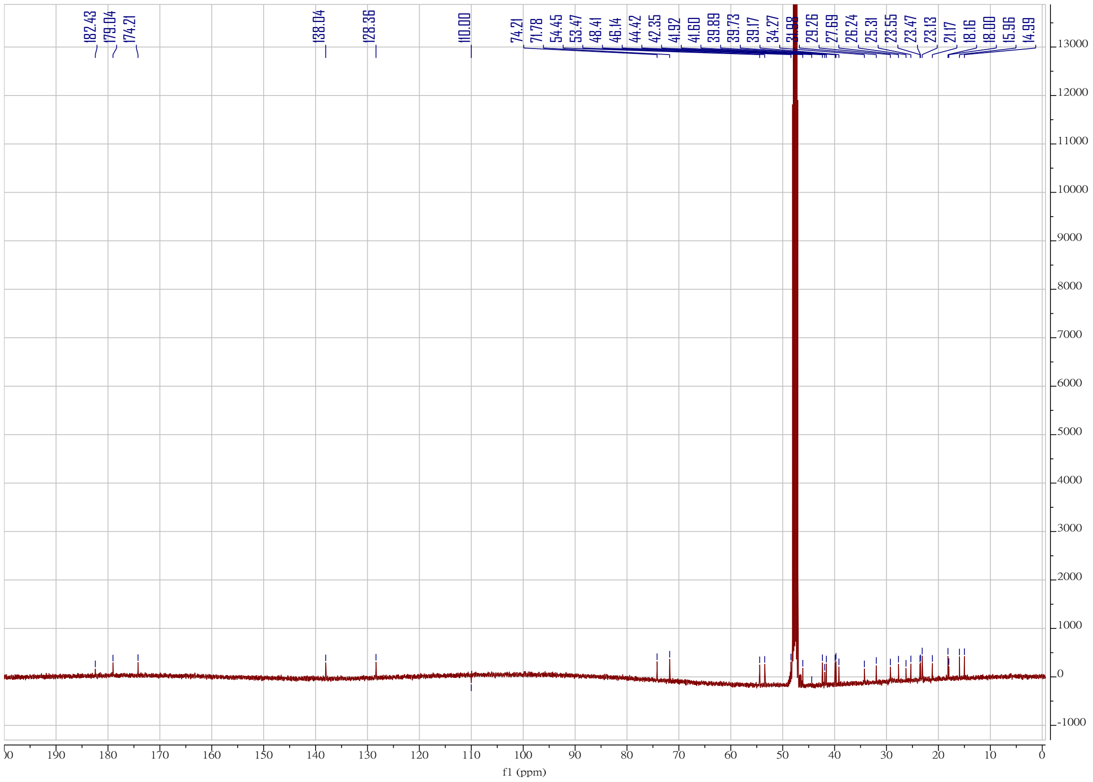

Figure S5.2. ^{13}C-NMR spectrum of compound **5** in methanol-d_4 (125 MHz).

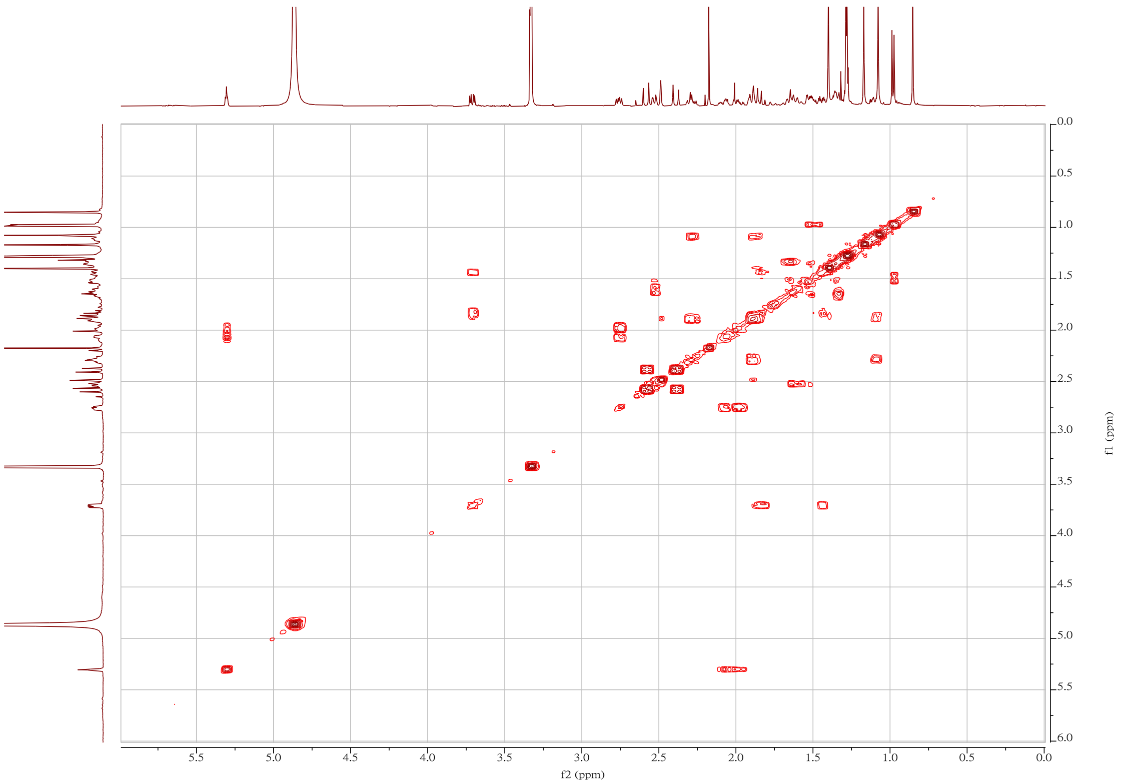

Figure S5.3. ^{1}H-^{1}H spectrum of compound **5**.

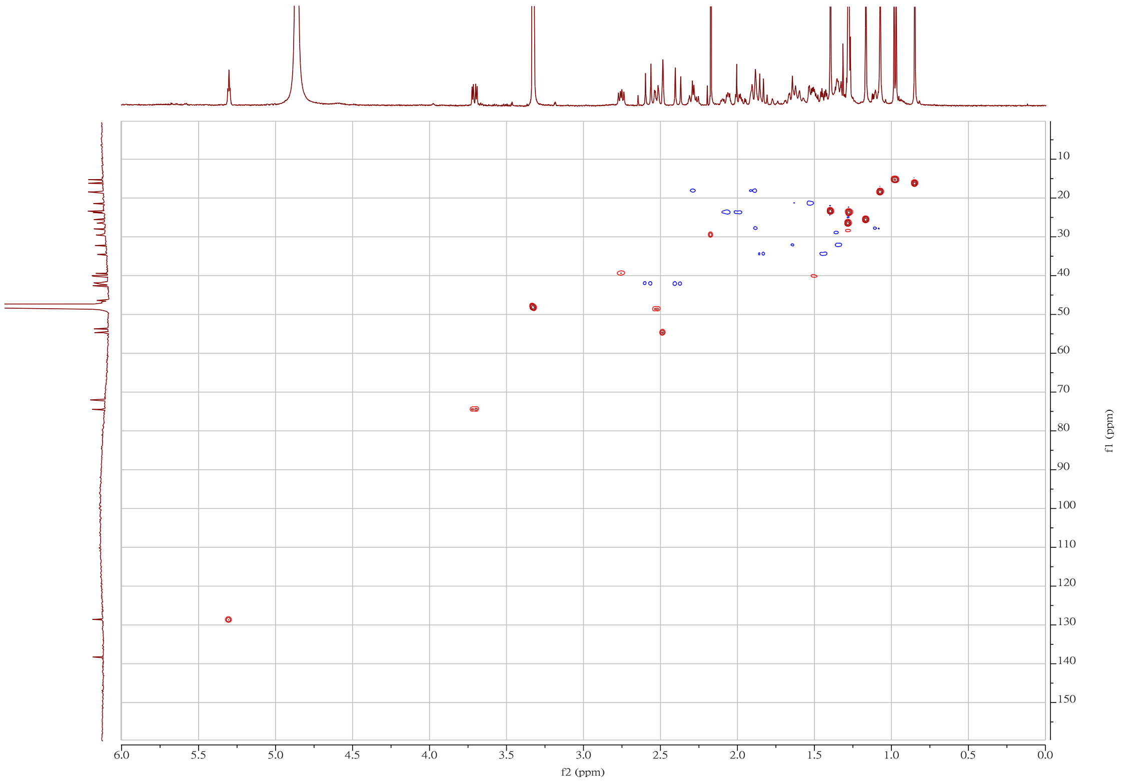

Figure S5.4. HSQC spectrum of compound **5**.

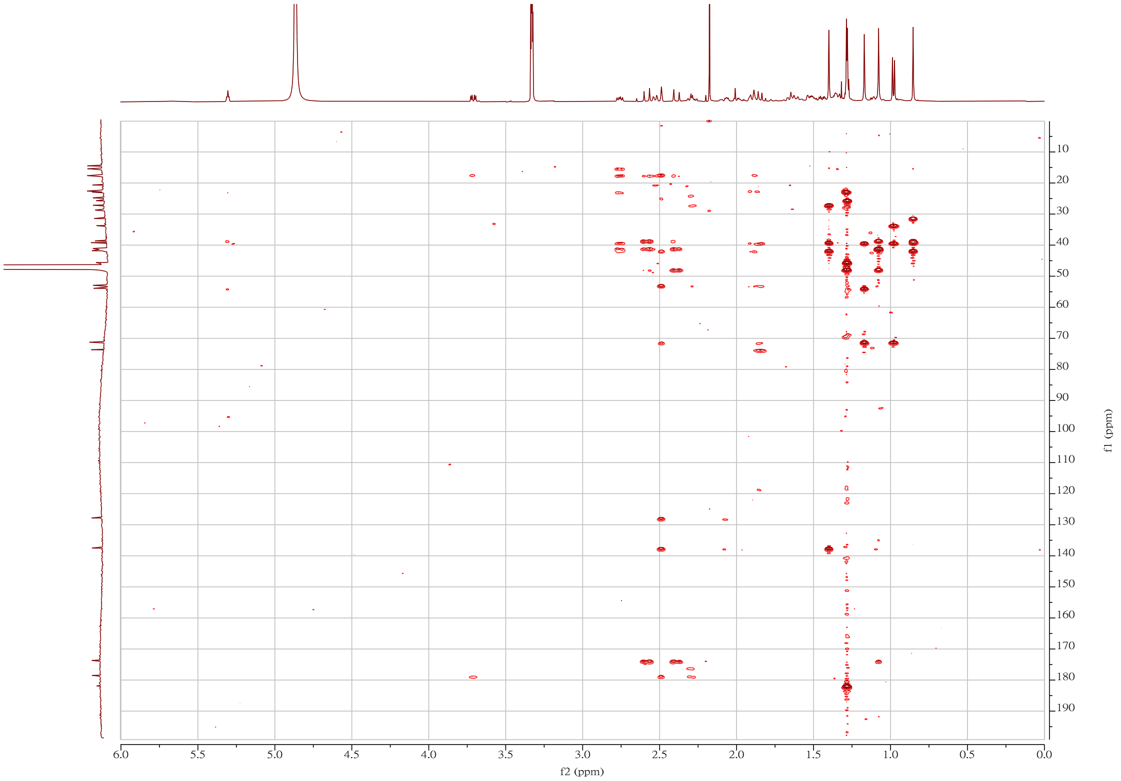

Figure S5.5. HMBC spectrum of compound **5**.

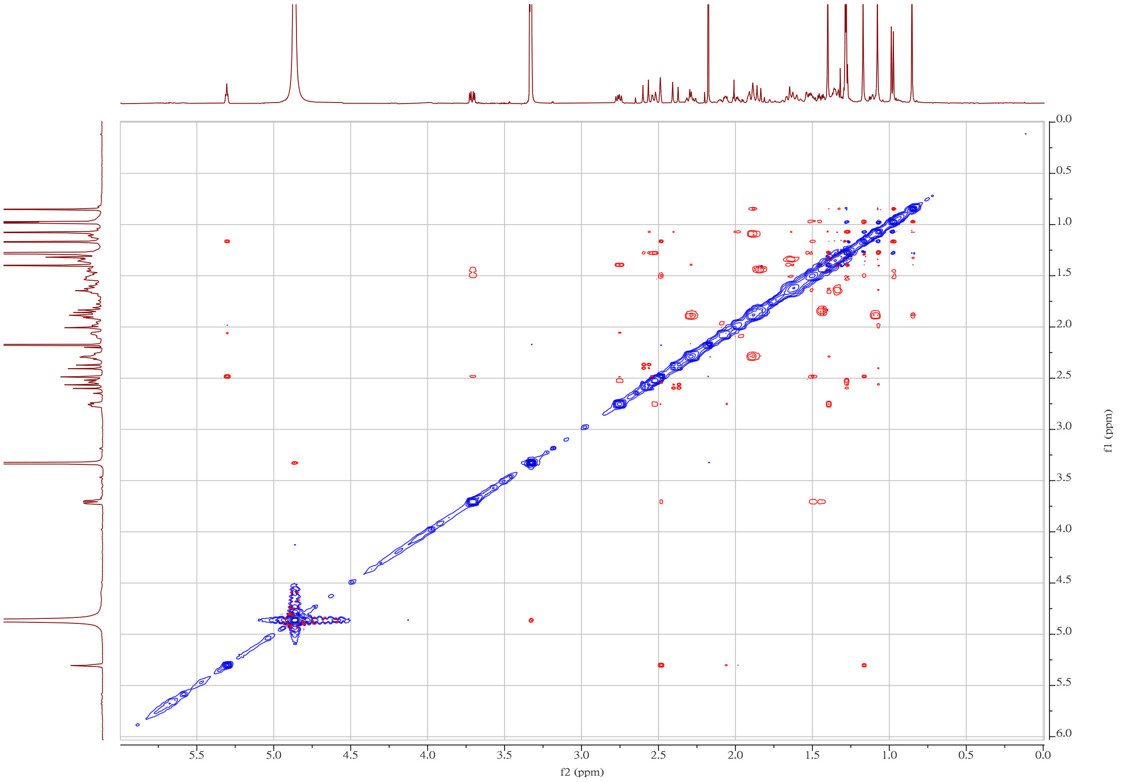

Figure S5.6. NOESY spectrum of compound **5**.

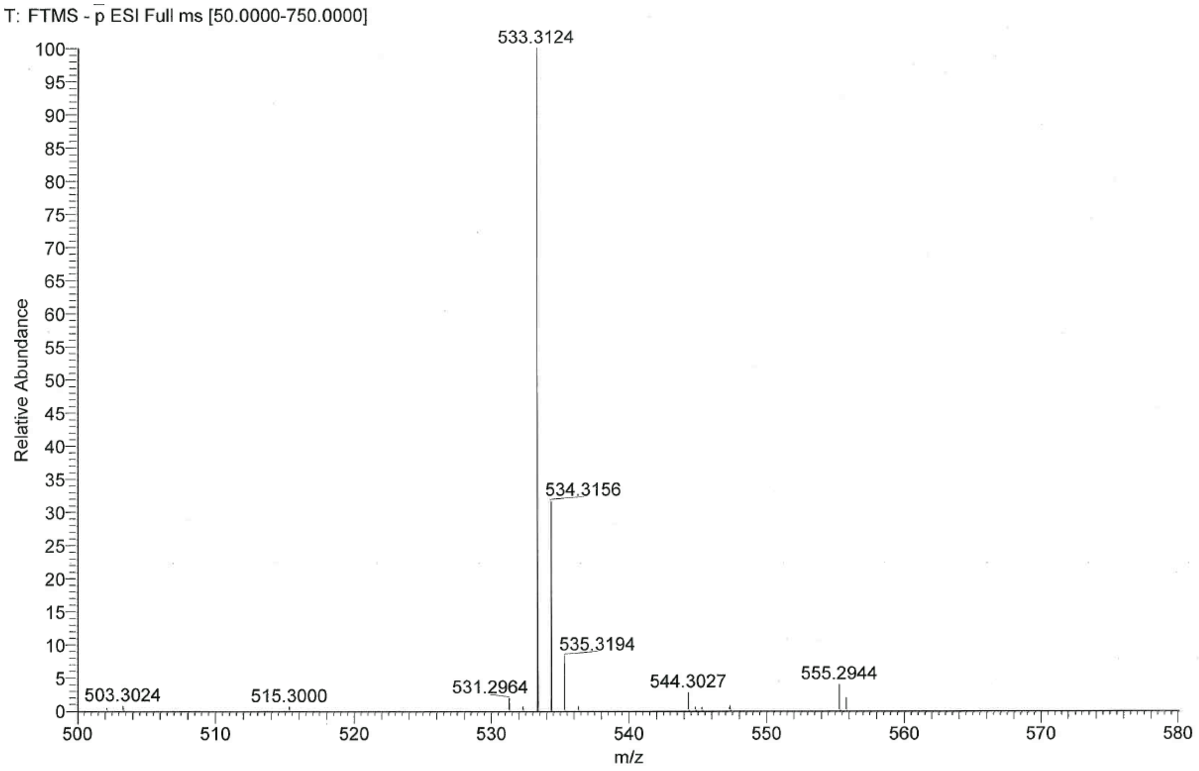

Figure S5.7. HRESIMS of compound **5**.

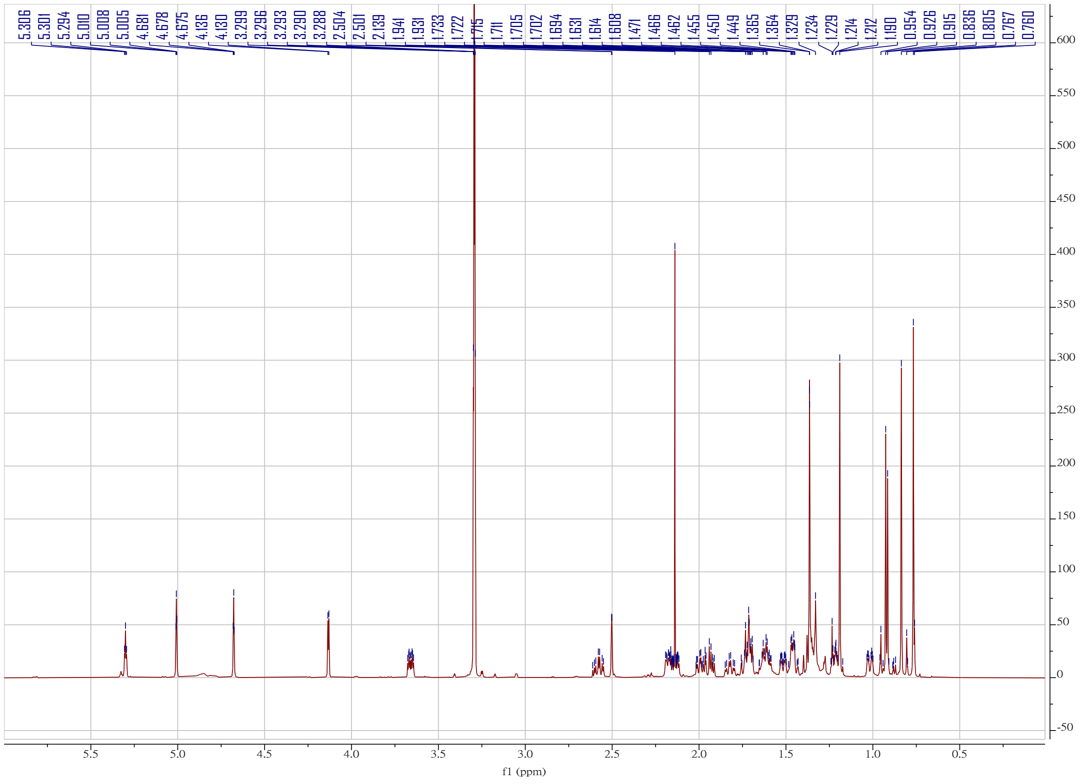

Figure S6.1. ^{1}H-NMR spectrum of compound **6** in methanol-d_4 (600 MHz).

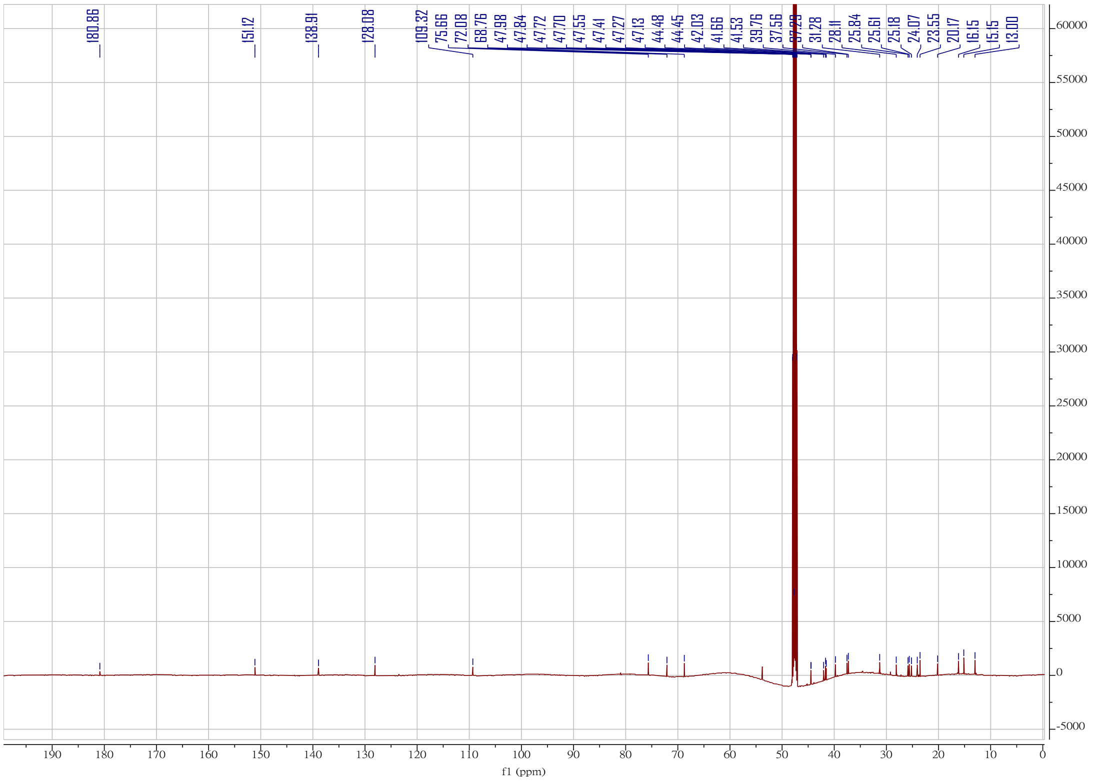

Figure S6.2. ^{13}C-NMR spectrum of compound **6** in methanol-d_4 (150 MHz).

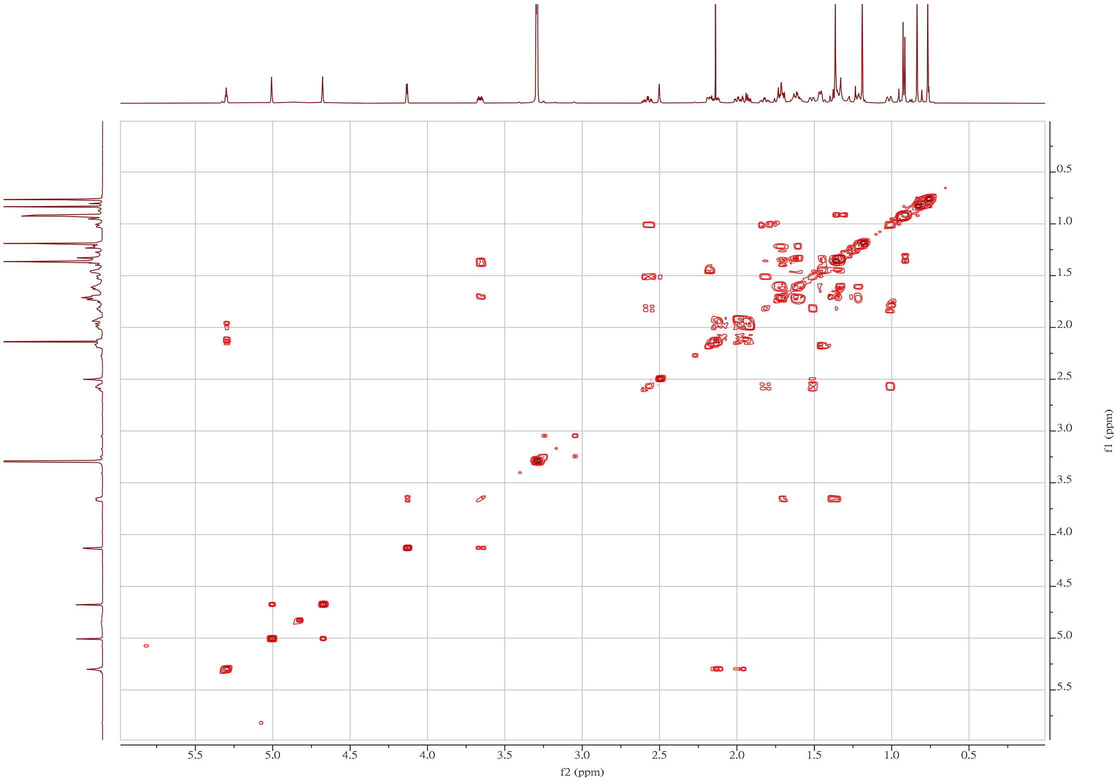

Figure S6.3. ^{1}H-^{1}H spectrum of compound **6**.

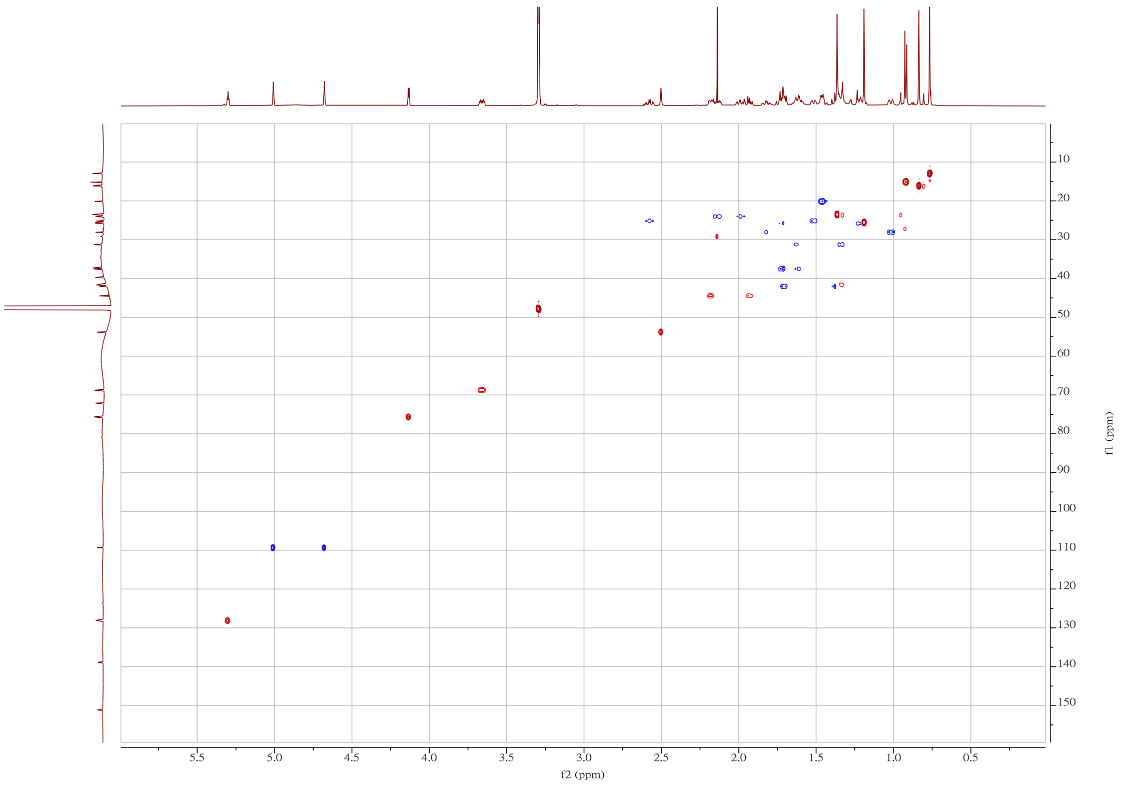

Figure S6.4. HSQC spectrum of compound **6**.

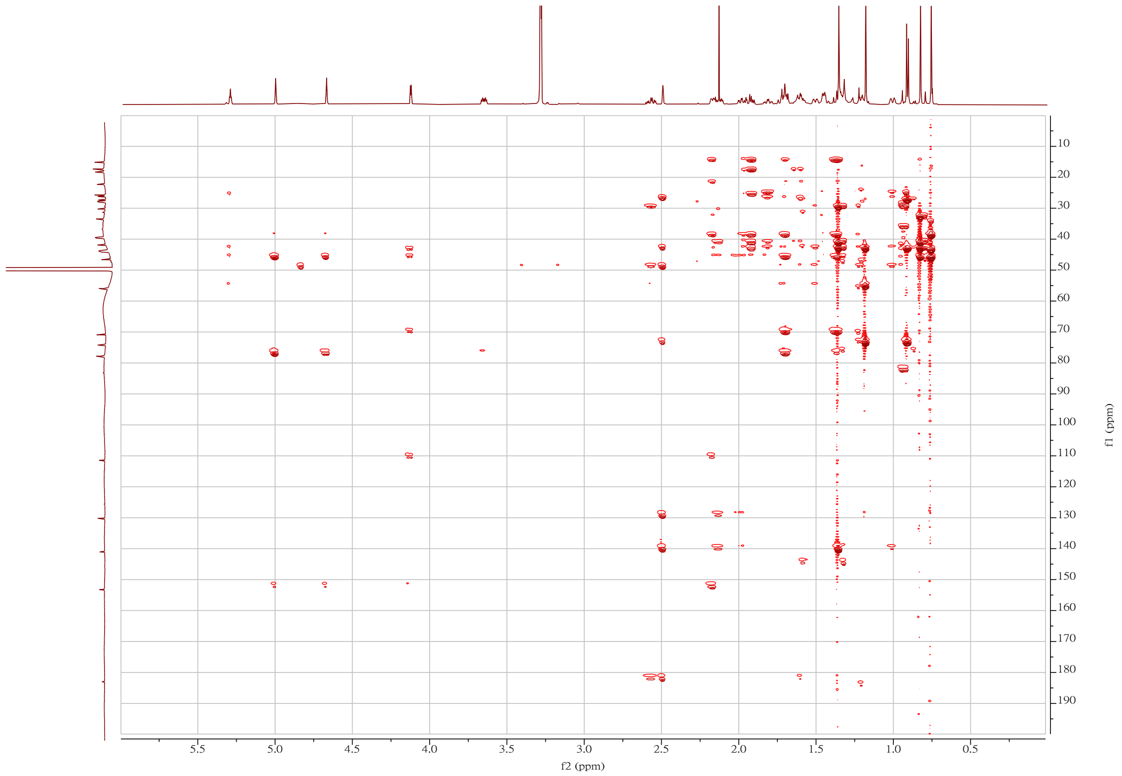

Figure S6.5. HMBC spectrum of compound **6**.

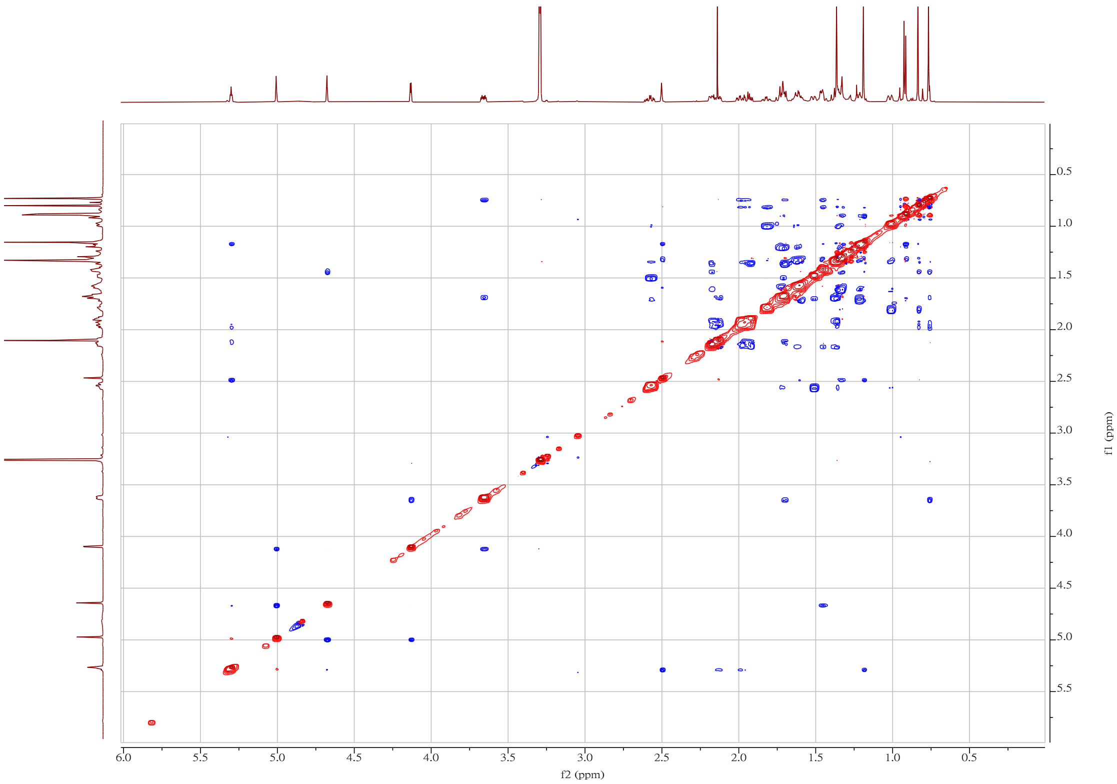

Figure S6.6. NOESY spectrum of compound **6**.

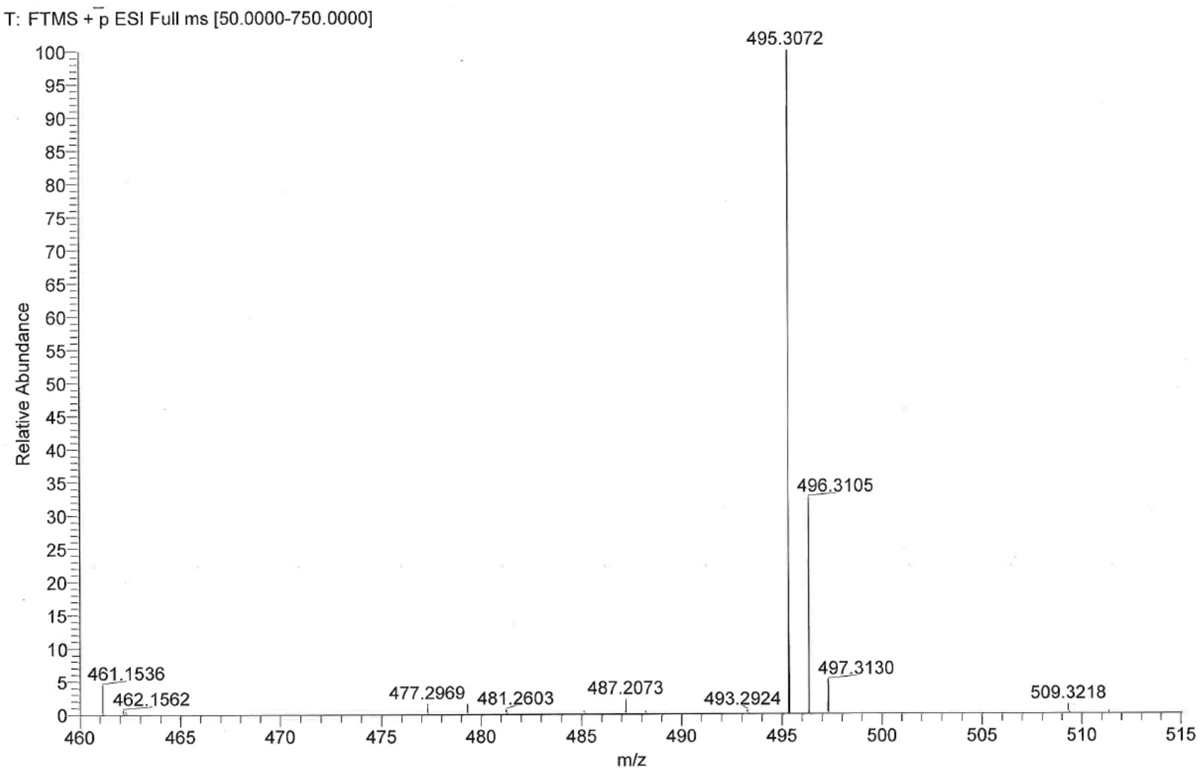

Figure S6.7. HRESIMS of compound **6**.

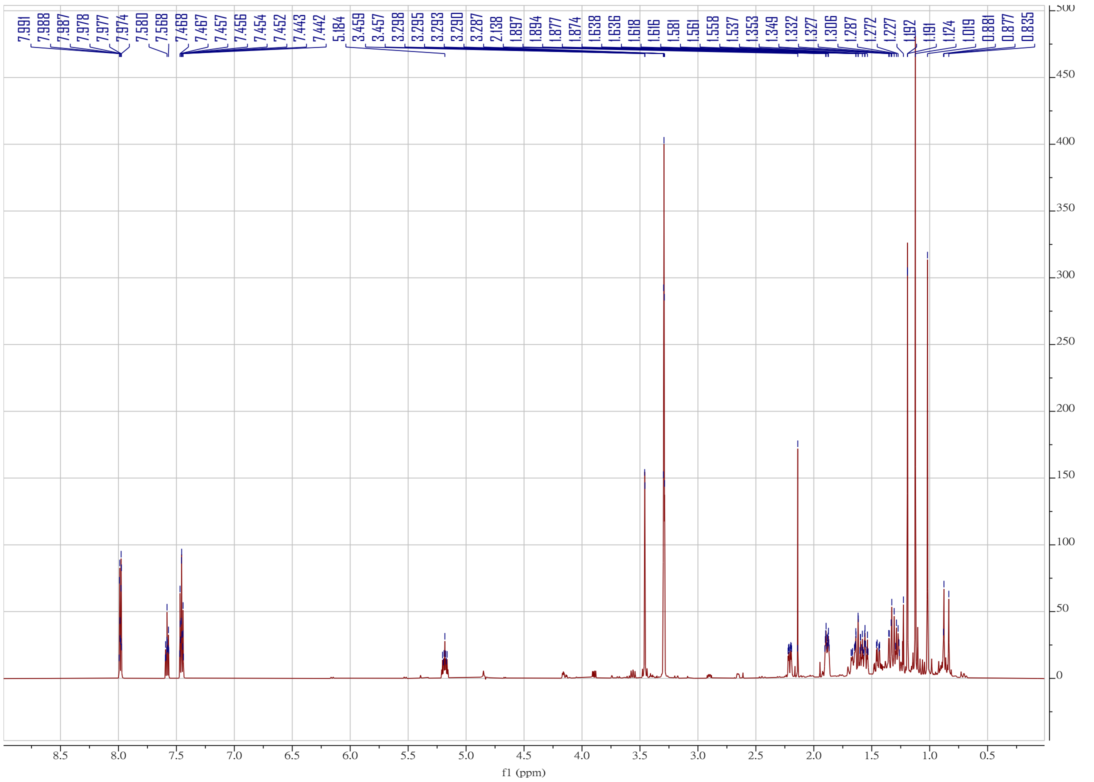

Figure S7.1. ^{1}H-NMR spectrum of compound **7** in methanol-d_4 (600 MHz).

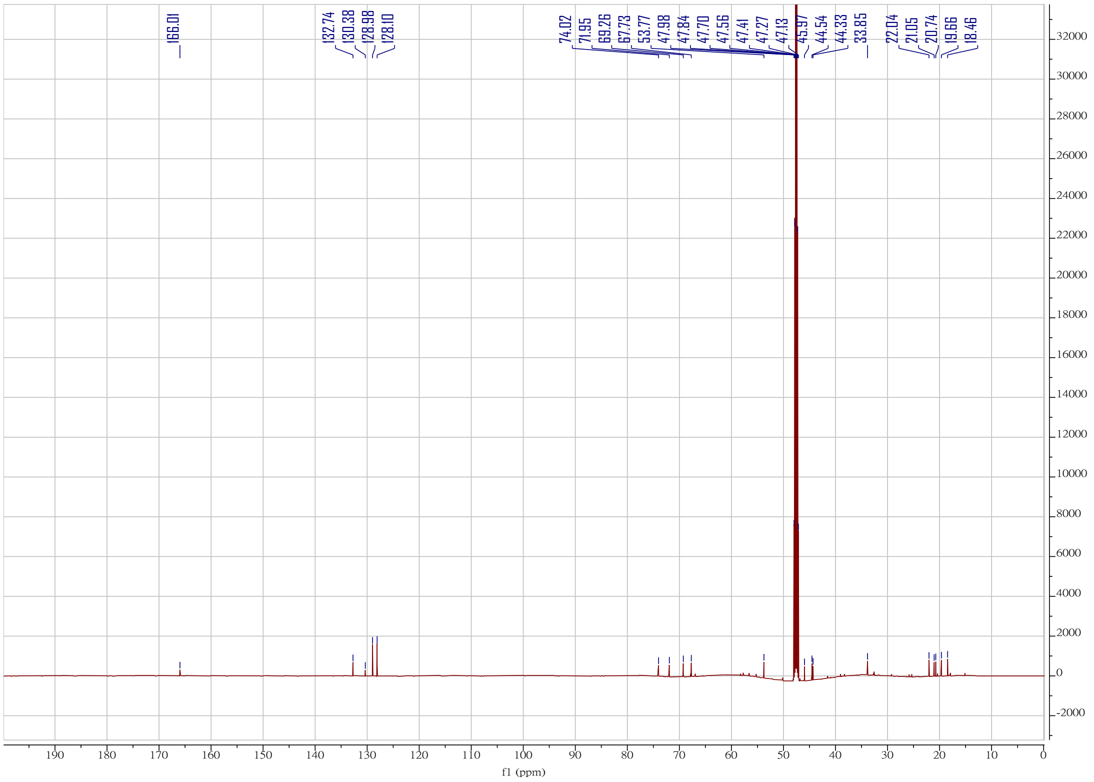

Figure S7.2. ^{13}C-NMR spectrum of compound **7** in methanol-d_4 (150 MHz).

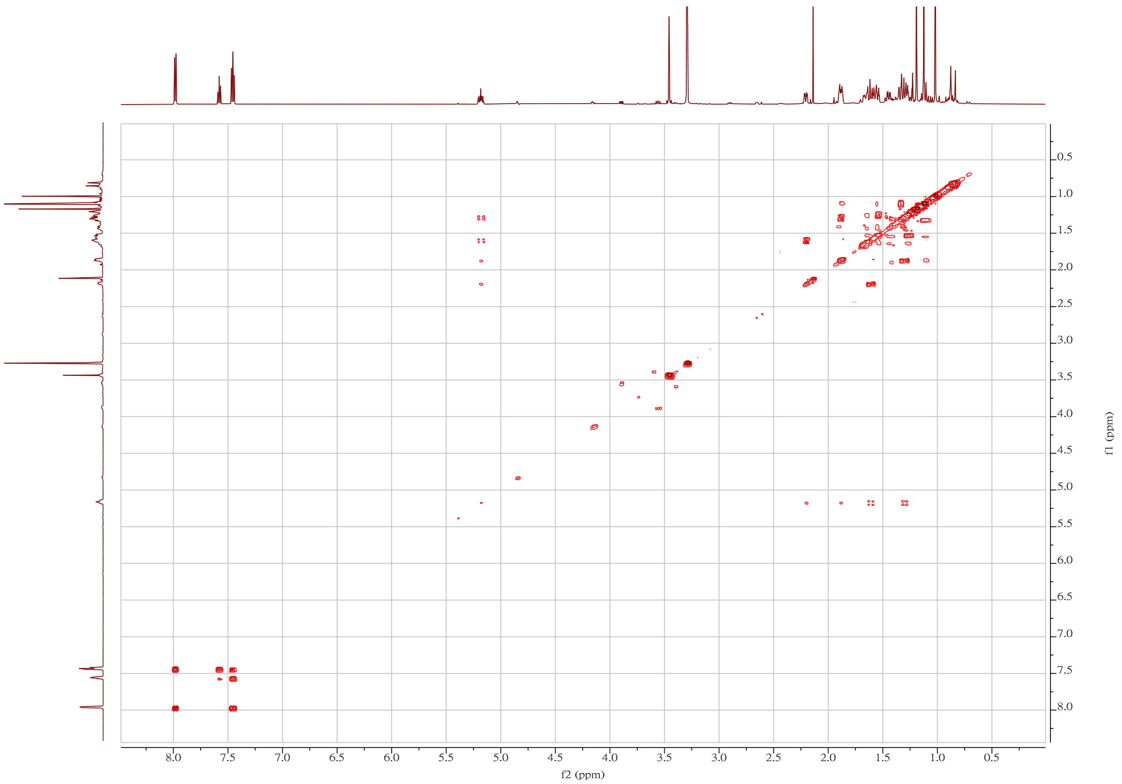

Figure S7.3. ^{1}H-^{1}H spectrum of compound **7**.

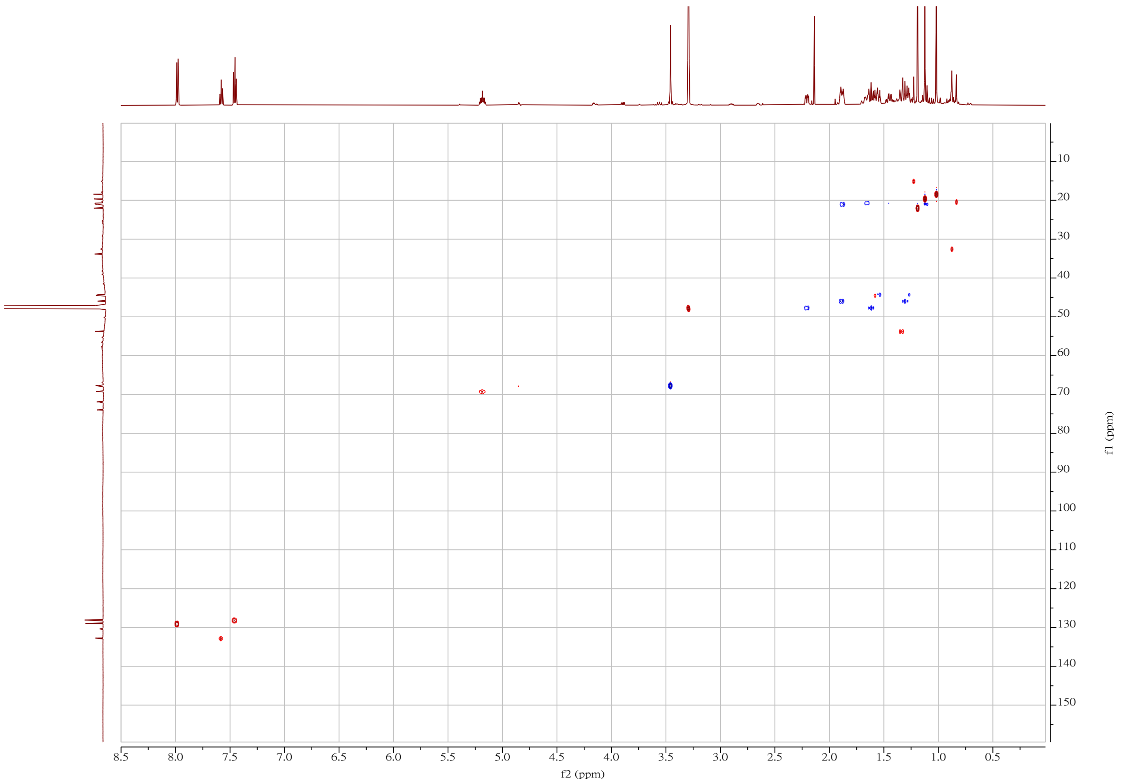

Figure S7.4. HSQC spectrum of compound **7**.

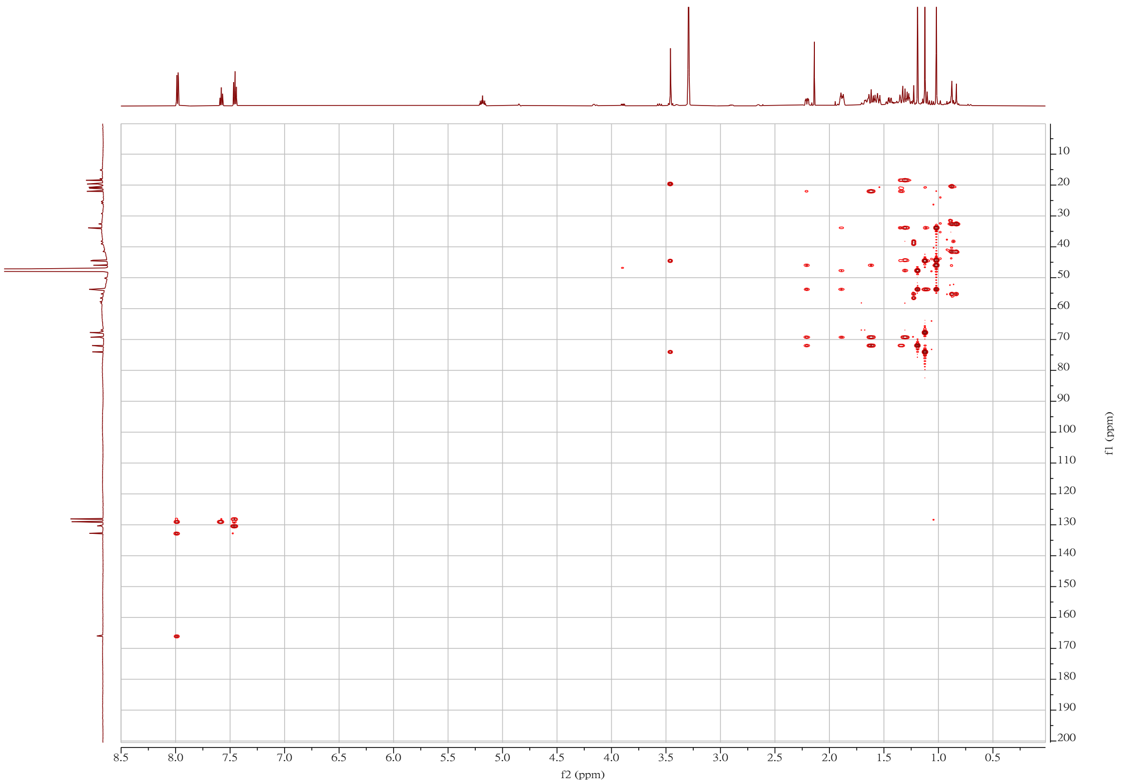

Figure S7.5. HMBC spectrum of compound **7**.

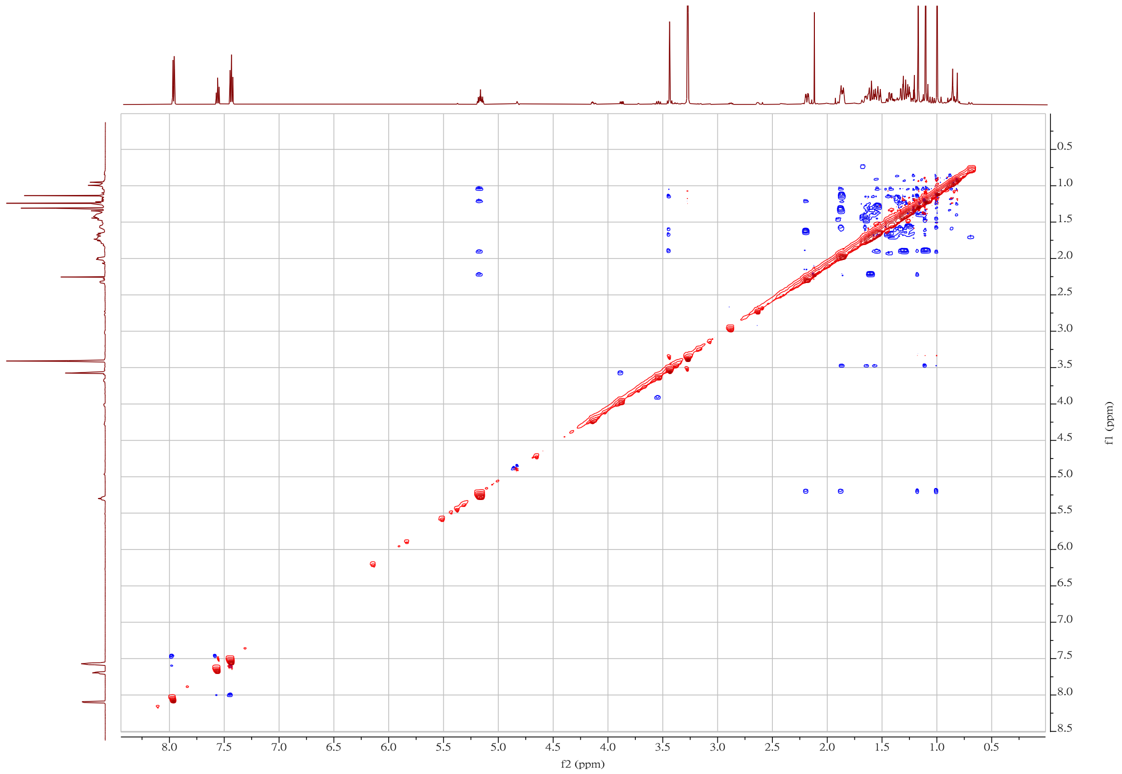

Figure S7.6. NOESY spectrum of compound **7**.

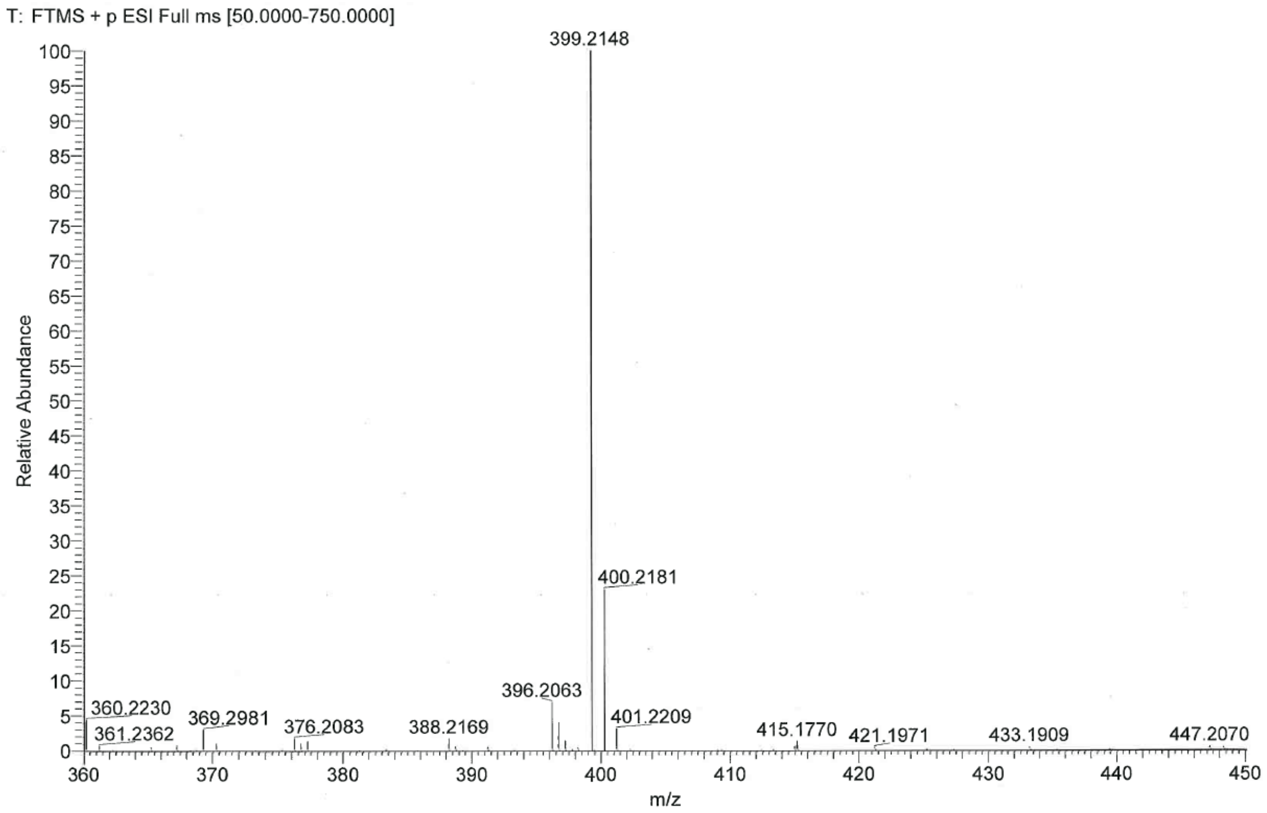

Figure S7.7. HRESIMS of compound **7**.
